# Mechanism Analysis of Antimicrobial Peptide NoPv1 Related to Potato Late Blight through a Computer-Aided Study

**DOI:** 10.3390/ijms25105312

**Published:** 2024-05-13

**Authors:** Jiao-Shuai Zhou, Hong-Liang Wen, Ming-Jia Yu

**Affiliations:** 1 School of Chemistry and Chemical Engineering, Beijing Institute of Technology, Beijing 100081, China; zjs2021@bit.edu.cn; 2 Key Laboratory of Medical Molecule Science and Pharmaceutical Engineering, Yangtze Delta Region Academy, Beijing Institute of Technology, Jiaxing 314019, China

**Keywords:** *Phytophthora infestans*, potato, biopesticide, molecular docking, dynamic simulation

## Abstract

*Phytophthora infestans* (Mont.) de Bary, the oomycotic pathogen responsible for potato late blight, is the most devastating disease of potato production. The primary pesticides used to control oomycosis are phenyl amide fungicides, which cause environmental pollution and toxic residues harmful to both human and animal health. To address this, an antimicrobial peptide, NoPv1, has been screened to target *Plasmopara viticola* cellulose synthase 2 (PvCesA2) to inhibit the growth of *Phytophthora infestans* (*P. infestans*). In this study, we employed AlphaFold2 to predict the three-dimensional structure of PvCesA2 along with NoPv peptides. Subsequently, utilizing computational methods, we dissected the interaction mechanism between PvCesA2 and these peptides. Based on this analysis, we performed a saturation mutation of NoPv1 and successfully obtained the double mutants DP1 and DP2 with a higher affinity for PvCesA2. Meanwhile, dynamics simulations revealed that both DP1 and DP2 utilize a mechanism akin to the barrel-stave model for penetrating the cell membrane. Furthermore, the predicted results showed that the antimicrobial activity of DP1 was superior to that of NoPv1 without being toxic to human cells. These findings may offer insights for advancing the development of eco-friendly pesticides targeting various oomycete diseases, including late blight.

## 1. Introduction

The potato, belonging to the Solanaceae family, is the world’s fourth-largest food crop after rice, wheat, and maize and plays a crucial role in global food security [1]. In 2020, potato production was estimated to be over 359 million tons, according to the Food and Agriculture Organization [2]. However, potato production is under serious threat from the disease of late blight, a disease caused by *Phytophthora infestans* (Mont.) de Bary, an oomycete pathogen, which is the most devastating factor affecting potato production [3]. The repeated and intensive use of pesticides, mainly phenyl amide fungicides, for controlling oomycete diseases in potatoes for many decades has led to environmental pollution, toxic residues with harmful effects on human and animal health, and the development of resistance in target pathogens [4]. Consequently, new bio-sustainable solutions need to be explored to take the place of conventional pesticides.

Biological studies on *Phytophthora infestans*, the causal agent of late blight in potatoes, provide valuable information on searching for targeting proteins in the discovery of the molecular mechanisms underlying oomycete plant infection [5]. Cellulose, the main component of *P. infestans*’ cell wall, accounts for 33.6% of the total glucan composition (85.6%) [6]. It appears to be essential for appressorium formation and efficient potato infection by *P. infestans*, as inhibition of its biosynthesis results in a significant reduction in normal germ tubes with appressoria, severe disruption of the cell wall in the pre-infection structures, and a complete loss of pathogenicity. Additionally, cellulose synthase gene expression is upregulated during the pre- and early-infection stages of *P. infestans* in potatoes [7]. Since cellulose is a vital component of *P. infestans*, targeting cellulose synthase represents a potential breakthrough in controlling *P. infestans*. Moreover, *P. infestans* cellulose synthase enzymes have been identified as the target of the fungicide mandipropamid, highlighting the importance of cell wall biosynthesis in oomycete disease development [8]. Colombo targeted *Plasmopara viticola cellulose synthase 2* (PvCesA2) and constructed an 8-amino acid length peptide library, successfully screening out peptide NoPv1 with potential inhibitory activity against *P. infestans* [9,10]. Other peptides, including NoPv2, NoPv3, NoPv1R1A, NoPv1R1AR7A, and NoPv1R7A, were also tested. In this study, we employed computer-assisted methods to investigate the binding modes between PvCesA2 and a series of NoPv peptides. Based on these findings, we want to investigate whether mutations within the core sequence can boost affinity. To achieve this, we conducted single- and double-point saturation mutagenesis on the eight amino acids using the DISCOVERY STUDIO v4.5 computer program. This involved mutating each amino acid into 19 different alternatives. These variants yield crucial insights into the correlation between structure and activity at the interface, assisting in designing antimicrobial peptides with diminished binding energy. According to these results, the structural models of single and double mutants with the lowest binding energy were built, and their relative affinities were analyzed by YASARA. The double mutants DP1 and DP2, which exhibited higher affinity with PvCesA2, were selected. This affinity was based on the binding mechanism between these designed peptides and PvCesA2, as revealed by molecular simulation. Following this, dynamic simulations were conducted to clarify the transmembrane mechanisms of DP1 and DP2. The findings suggest that both DP1 and DP2 employ a mechanism akin to the barrel-stave model for penetrating the cell membrane.

## 2. Results and Discussion

### 2.1. Characterization of the Receptor Model

**Figure 1:**
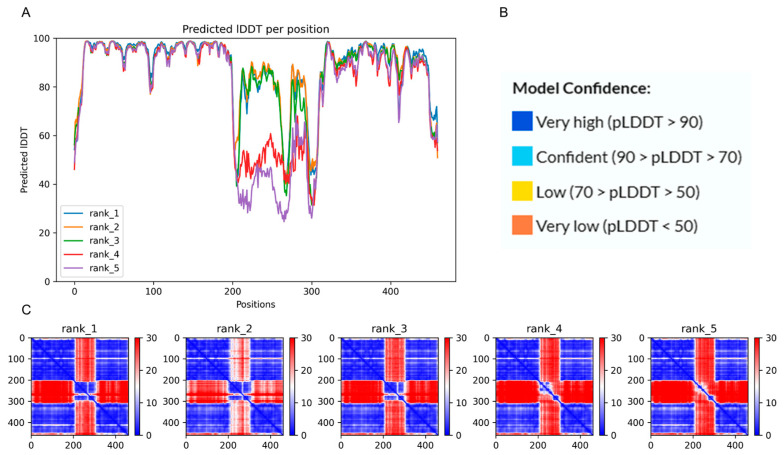
PvCesA2 homology models in detail. (**A**) pLDDT per position. (**B**) Measurement of model confidence. This section presents the quality of the model alongside its corresponding pLDDT score. (**C**) The predicted aligned error (PAE) is visually represented for each structural prediction.

The 3D homology structure of PvCesA2 has 460 amino acids and consists of chain A, which was utilized in this study. The maximum score achieved in the local distance difference test (pLDDT) was 88.1, as displayed in Table 1, signifying the model’s acceptable quality. Details of PvCesA2 homology modeling were shown in Figure 1.

### 2.2. Characterization of the Ligand Models

**Figure 2:**
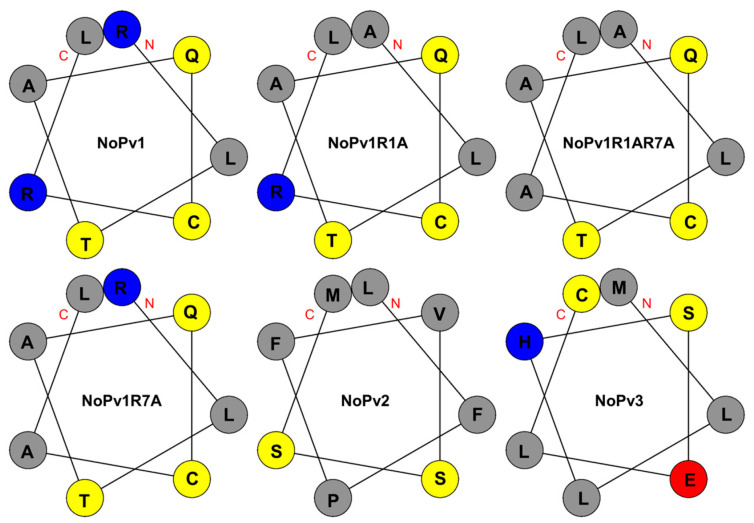
Helix-wheel analysis of the peptides. Polar neutral amino acids are shown in yellow; Basic residues are shown in blue; Acidic residues are shown in red; and Non-polar amino acids are shown in grey. The peptide’s head is denoted by the red letter “N”, whereas its tail is marked by the red letter “C”.

Each of the 3D structures of the NoPv peptides has 8 amino acids based on homologous modeling. The partial parameters of the NoPv peptides and their respective highest pLDDT scores are shown in Table 2. The Grand average of hydropathicity (GRAVY) of peptides, calculated based on the Kyte-Doolittle scale, is determined by summing the hydropathy values of all amino acids and then dividing by the total number of residues in the sequence. A higher positive score indicates greater hydrophobicity [11]. Additionally, the charge is the total net charge of the peptides, calculated from the APD3 website [12]. Helical wheel analysis (http://www.bioinformatics.com.cn, accessed on 9 August 2023) [13] was calculated to show the distribution of polar and non-polar amino acids in NoPv peptides (Figure 2). Accordingly, based on the parent peptide NoPv1, which has two positive charges, the positively charged arginine (R) residues at the N- and C-terminus were sequentially substituted by the hydrophobic alanine (A) residues, which generated NoPv1R1A, NoPv1R1AR7A, and NoPv1R7A with different hydrophobicity and charges (Table 2). Among them, NoPv1R1A and NoPv1R7A possessed identical net charges (+1), but that of NoPv1R1AR7A was different (0). Meanwhile, as the number of alanine (A) substitutions increased, the hydrophobicity of the peptide derivatives gradually rose, as indicated by the increasing GRAVY values. Moreover, NoPv2 and NoPv3 showed equivalent net charges, although the former did not contain polar-charged amino acids while the latter possessed an equal number of basic (H) and acidic (E) amino acids. Compared to NoPv1, the hydrophobicity of NoPv2 and NoPv3 was found to increase due to the possession of more hydrophobic amino acids.

### 2.3. Analysis of Molecular Interactions by Molecular Modeling

**Figure 3:**
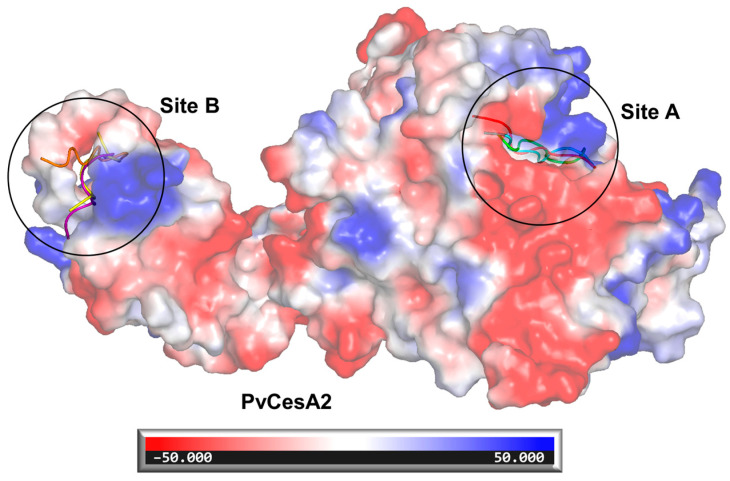
Electrostatic surface and the binding sites of NoPvs docked in PvCesA2. A. NoPv1, NoPv1R1A, and NoPv1R1AR7A bind on site A; B. NoPv2, NoPv3, and NoPv1R7A bind on site B. NoPv1 is shown in red, NoPv1R1A is shown in green, NoPv1R1AR7A is shown in cyan, NoPv2 is shown in yellow, NoPv3 is shown in orange, and NoPv1R7A is shown in purple. Blue surfaces represent electropositive surfaces, while red surfaces represent electronegative surfaces.

The results of the molecular docking between the NoPv peptides and PvCesA2 in Table 3 showed that NoPv1 bound more tightly (highest binding affinity) to PvCesA2 (binding energy and predicted dissociation constant of −6.949 kcal/mol and 8.060 μM) than that of other NoPv peptides. Overlay of the complexes showed that the NoPv peptides bound to PvCesA2 at different sites (Figure 3).

**Figure 4:**
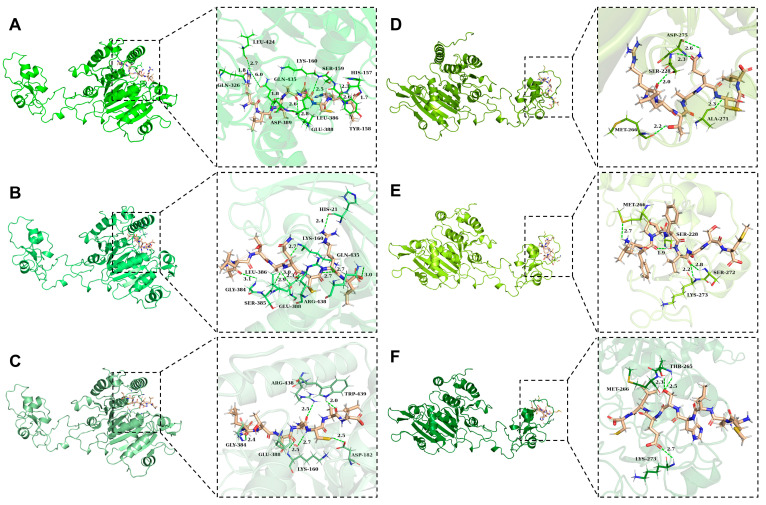
Interactions of the NoPv peptides with PvCesA2. (**A**) Interaction between NoPv1 and PvCesA2. (**B**) Interaction between NoPv1R1A and PvCesA2. (**C**) Interaction between NoPvR1AR7A and PvCesA2. (**D**) Interaction between NoPv1R7A and PvCesA2. (**E**) Interaction between NoPv2 and PvCesA2. (**F**) Interaction between NoPv3 and PvCesA2. The green dashed lines represent conventional hydrogen bonds.

NoPv1, NoPv1R1A, and NoPv1R1AR7A bind to PvCesA2 on site A (Figure 3), with the amino acids LYS-160, GLU-388, and ASP-438 on the surface of PvCesA2 forming hydrogen bonds stably with T3, A4, Q5, and C6 in NoPv1, A4, Q5, C6, and R7 in NoPv1R1A, while A4, Q5, and C6 in NoPv1R1AR7A (Figure 4). LYS-160, ALA-364, and TRP-439 on the surface of PvCesA2 interact with steady hydrophobic contact with R1, L2, and A4 in NoPv1, R7, and L8 in NoPv1R1A, while A4, R7, and L8 are in NoPv1R1AR7A. The ligand-receptor hydrogen bond interactions are recognized as pivotal contributors to binding owing to their specificity, while hydrophobic interactions also significantly contribute to protein stability [14]. It is evident that hydrogen bonds play a key role in the binding between the NoPv peptides and PvCesA2 (Tables S1–S3). The variation in the total number of hydrogen bonds suggests that a higher number of hydrogen bonds correlates with stronger binding affinity (−6.949 kcal/mol for NoPv1, −6.567 kcal/mol for NoPv1R1A, and −6.268 kcal/mol for NoPv1R1AR7A). Likewise, NoPv1R7A, NoPv2, and NoPv3 bind to PvCesA2 on site B (Figure 3), with the amino acids SER-228, ASN-263, MET-266, and LYS-273 on the surface of PvCesA2 forming hydrogen bonds stably with R1, L2, T3, and Q5 in NoPv1R7A, L1, F2, V5, S6, and S7 in NoPv2, while L3, H4, S5, E6, and L7 in NoPv3 (Figure 4). ILE-231 on the surface of PvCesA2 interacts with steady hydrophobic contact with R1 and L2 in NoPv1R7A, P3 in NoPv2, and L7 and C8 in NoPv3. In particular, NoPv3 has a lower number of hydrogen bonds, and the binding affinity to PvCesA2 (−4.744 kcal/mol) is much smaller than that of NoPv2 (−6.334 kcal/mol) and NoPv1R7A (−6.310 kcal/mol) (Tables S4–S6).

Above all, the binding affinity results of six NoPv peptides showed that the huge difference in binding sites directly affects the interactions on the interface between PvCesA2 and its ligands. Particularly among the peptides examined, NoPv1 emerges as the most effective inhibitor at the binding site of PvCesA2, forming the highest number of hydrogen bonds compared to the other peptides (Tables S1−S6). Moreover, NoPv1 with its derivatives binding on site A exhibited relatively higher binding energies, and site A would be a more ideal site for binding, where the ligand can exert a better effect to inhibit the enzyme activity.

### 2.4. Saturation Mutagenesis of NoPv1 In Silico

**Figure 5:**
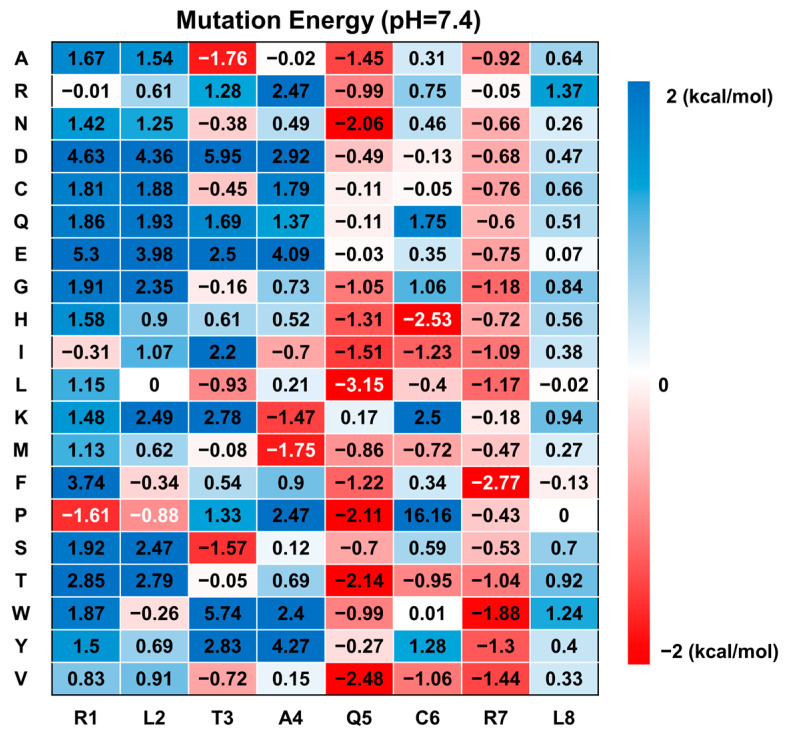
Predicted mutation energy of mutants for PvCesA2-NoPv1 complexes at pH 7.4.

**Figure 6:**
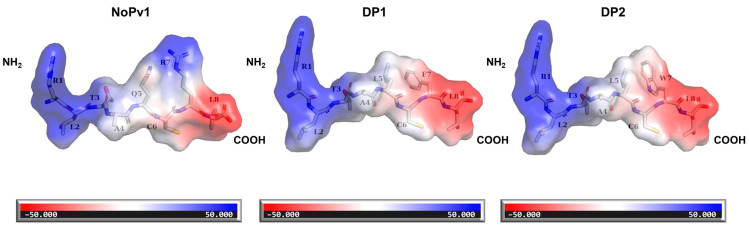
The electrostatic potential on the surface of peptides. Blue surfaces represent electropositive surfaces, while red surfaces represent electronegative surfaces.

Based on the computational simulation results, each amino acid in NoPv1 underwent mutation into 19 other amino acids, and the mutation energy for each PvCesA2-peptide complex was calculated using DISCOVERY STUDIO. The findings revealed that numerous single mutations led to lower mutation energy, suggesting a potential improvement in binding affinity. Notably, residues Q5 and R7 appeared to be unfavorable for binding with PvCesA2, as most mutations at these sites resulted in lower mutation energy (Figure 5). Thus, we also chose the double mutation of Q5 and R7 to do the saturation mutagenesis calculation (Figure S2). Above all, 9 mutated peptides, including 7 single mutants (SP1-7) and 2 double mutants (DP1-2), together with the wild-type (NoPv1) were selected, and their corresponding binding affinities were analyzed by YASARA (Table 4). Among all the single mutants, only SP5 and SP7 showed higher affinity to PvCesA2 (binding energies of −7.152 kcal/mol and −7.383 kcal/mol) than the native one, which is also consistent with the predicted mutation energy of the PvCesA2-NoPv1 complex (mutation energies of −3.15 kcal/mol and −2.77 kcal/mol). Therefore, we designed the double mutants DP1 and DP2, which have relatively higher affinities to PvCesA2 (binding energies of −7.564 kcal/mol and −7.551 kcal/mol) than SP5 and SP7. Additionally, the R1 residue at the N-terminus may be crucial for facilitating the cellular uptake of antimicrobial peptides, allowing them to reach the catalytically active region of PvCesA2, which is located intracellularly (Figure S3). This is in agreement with the mechanism of action of some cationic antimicrobial peptides, which have been reported to penetrate cells and affect cellular physiological processes without altering the permeability of microbial membranes [15]. Moreover, the polar amino acid R7 at the C-terminus in NoPv1 was mutated to non-polar amino acids, resulting in the absence of positive electrostatic potential near the C-terminus (Figure 6) and an increase in the binding affinity of the mutants. This indicates that non-polar amino acids, which act as skeleton support in the sequence, may be an important factor in determining how tightly the antimicrobial peptide binds to the target protein. During the mutation process, the ligand consistently bound within the active pocket composed of the amino acids HIS-157, TYR-158, SER-159, LYS-160, GLU-388, GLN-326, GLN-435, LEU-424, ASP-389, LEU-386, and TRP-439 (Tables S1, S7 and S8).

### 2.5. Dynamics Simulation Analysis

The molecular docking analysis offered insights into the structural basis of the binding mechanism of both native and mutated peptides. However, this method is theoretically coarse due to the relatively rigid model it employs. To validate the reliability of models generated from protein-protein docking, molecular dynamics (MD) simulations were conducted. These simulations assessed the structural stability of the complex by analyzing parameters such as the root-mean-square deviation (RMSD), protein-peptide interaction energy, root-mean-square fluctuation (RMSF), and radius of gyration (Rg) throughout the trajectory.

**Figure 7:**
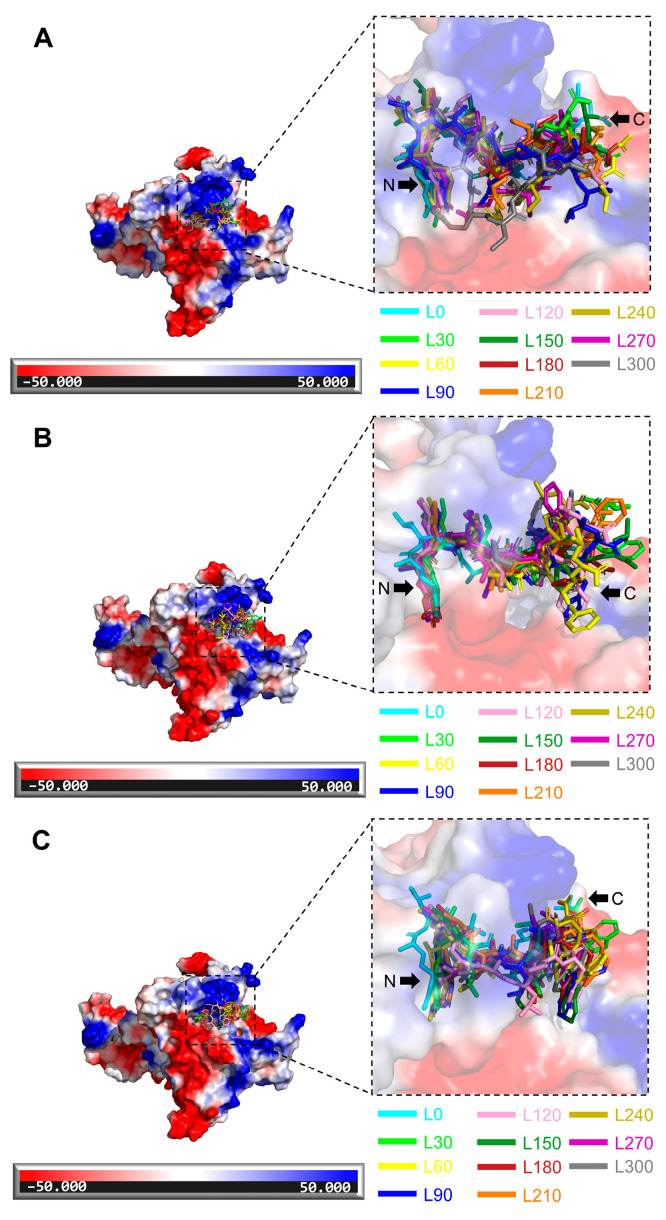
Electrostatic surface rendering of the PvCesA2-peptide complexes during the MD simulation. (**A**) NoPv1-PvCesA2. (**B**) DP1-PvCesA2. (**C**) DP2-PvCesA2. Blue surfaces represent electropositive surfaces, while red surfaces represent electronegative surfaces. L0, L30, L60, L90, L120, L150, L180, L210, L240, L270, and L300 are colored in cyan, carbon, dash, blue, pink, forest, firebrick, orange, olive, purple, and gray. (e.g., L30 represents the location of NoPv1/DP1/DP2 at 30 ns during the MD simulation period).

During the 300 ns dynamics simulation, we analyzed the changes in each complex and protein from their initial configuration to their final position. The RMSD profiles of all MD structures (Figure S4) indicated that the variation in RMSD values remained stable, generally staying below 1 Å. The binding energy distribution of the 300 ns (Figure S6) simulations also stabilized, and the binding energy of DP1 was overall higher than that of NoPv1 and DP2, which is consistent with the docking results. The RMSF profile (Figure S5) indicated minimal residual fluctuations in the active site region, and all complex systems exhibited stable conformational behavior in PvCesA2. Typically, higher RMSF values denote increased flexibility or decreased stability of the structures, whereas lower RMSF values signify enhanced stability of the structures [16]. Furthermore, the radius of gyration is a critical parameter for predicting the compactness behavior of proteins. The Rg plots (Figure S5) tend to stabilize after the 300 ns dynamic simulation. Greater variation in the Rg score results from conformational changes or protein folding, whereas lower fluctuation over the simulation time suggests higher compactness and rigidity of a structure [17]. Then, we overlaid the final structures obtained by MD with the original structures for all complexes. The RMSD values from PyMOL alignment, encompassing the final MD structure and the initial docking complex, are 2.015, 1.867, and 1.981 (<3 Å [18]), respectively (Figure S7), which indicated that the structural variances are minor. Furthermore, we intercepted a conformation every 30 ns and calculated the electrostatic potential map of the PvCesA2-peptide complexes (Figure 7). The red region indicates an electron-rich area with electrophilic activity and a negative electrostatic potential, whereas the blue region denotes an electron-deficient region with nucleophilic activity and a positive electrostatic potential [19]. It can be seen that the conformational positions of peptides deviate rarely in each time interval. We also selected the final MD structures of complexes for interaction analysis. Interestingly, we found that the amino acids GLN-326, ALA-364, and GLN-435 on the surface of PvCesA2 interact with NoPv1 and its mutants (Figure S8) in particular, the oxygen atoms on GLN-326 and ALA-364 of PvCesA2 consistently form hydrogen bonds, interacting with the hydrogen atom on R1 of NoPv1 and its mutants located at the N-terminus (Tables S9–S11). It is worth noting that the mutation of NoPv1 to DP1/DP2 does not affect the hydrogen bonding effect of amino acid GLN-326 as well as the hydrophobic contact of amino acid TRP-439 on the surface of PvCesA2 throughout the MD simulation (Tables S9–S11).

### 2.6. Frontier Molecular Orbitals Analysis

**Figure 8:**
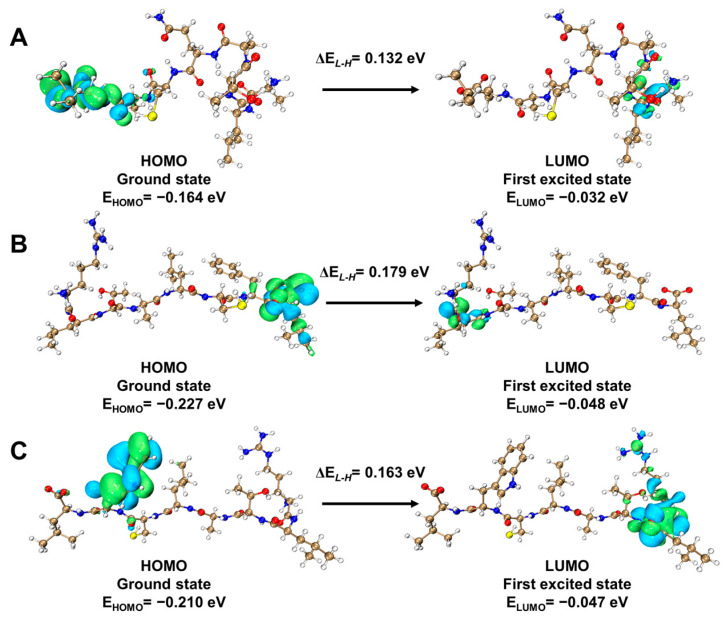
The FMOs including HOMO and LUMO for (**A**) NoPv1, (**B**) DP1, (**C**) DP2, as calculated at the B3LYP-D3/6-31+G(d,p) level of DFT. The positive and negative phases of the orbital wave function are shown in light green and light blue respectively.

The frontier molecular orbitals (FMOs) are usually referred to as the highest occupied molecular orbital (HOMO) and lowest unoccupied molecular orbital (LUMO). The structural characteristics of ligands employed in docking interactions with selected protein molecules play a crucial role in elucidating the density distribution pattern on frontier molecular orbitals [20]. We conducted FMOs analysis on NoPv1, DP1, and DP2. The electronic and structural properties of peptides were computed by optimizing their structures using the B3LYP-D3/6-31+G(d,p) density functional theory level. The HOMO-LUMO energy difference (∆*E*_*L*-*H*_) is crucial for assessing the molecule’s stability and reactivity [21]. Outstanding molecule inhibitors are capable of accepting and donating free electrons to a vacant orbital, thereby increasing their electron richness and ultimately providing superior inhibition efficiency [22]. The energetic outcomes of FMOs indicated that the ∆*E*_*L*-*H*_ values of the HOMO-LUMO difference for NoPv1, DP1, and DP2 are 0.132 eV, 0.179 eV, and 0.163 eV, respectively. This suggests that DP1 and DP2 facilitate higher charge transmission, forming stable interactions with the target protein, thereby enhancing bioactivity compared to NoPv1, as displayed in Table 5 and Figure 8.

### 2.7. Transmembrane Mechanism of DP1 and DP2

While the cell wall aids substance movement between the cell and its surroundings, it also allows antimicrobial peptides to enter the periplasmic space, or the region adjacent to the cell membrane, in specific organisms [23]. Although the cell membrane acts as the primary barrier against peptide entry into the cell interior, it must overcome its defenses to access intracellular targets and carry out antimicrobial functions. To understand the transmembrane mechanism of DP1 and DP2 at the molecular level, all-atom molecular dynamics simulations were conducted.

**Figure 9:**
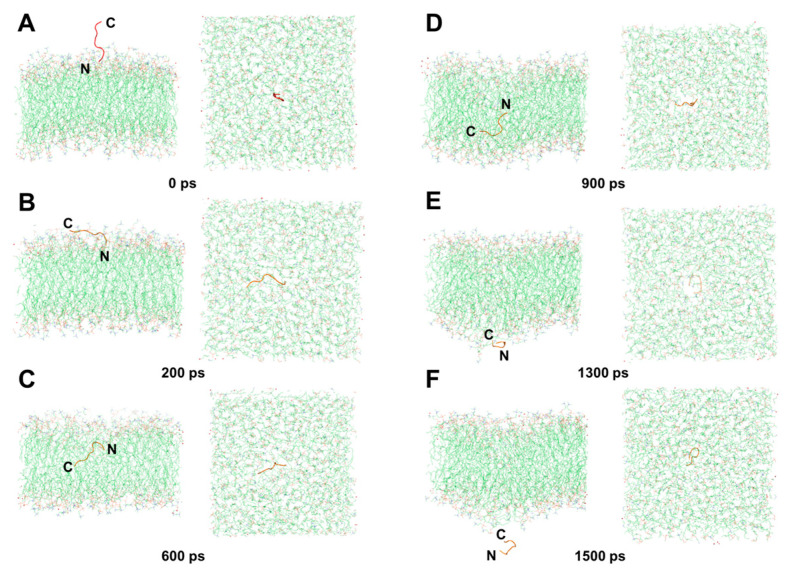
Translocation process diagram of the DP1, with the N-terminus approaching the surface of the cell membrane. (**A**) 0 ps. (**B**) 200 ps. (**C**) 600 ps. (**D**) 900 ps. (**E**) 1300 ps. (**F**) 1500 ps.

**Figure 10:**
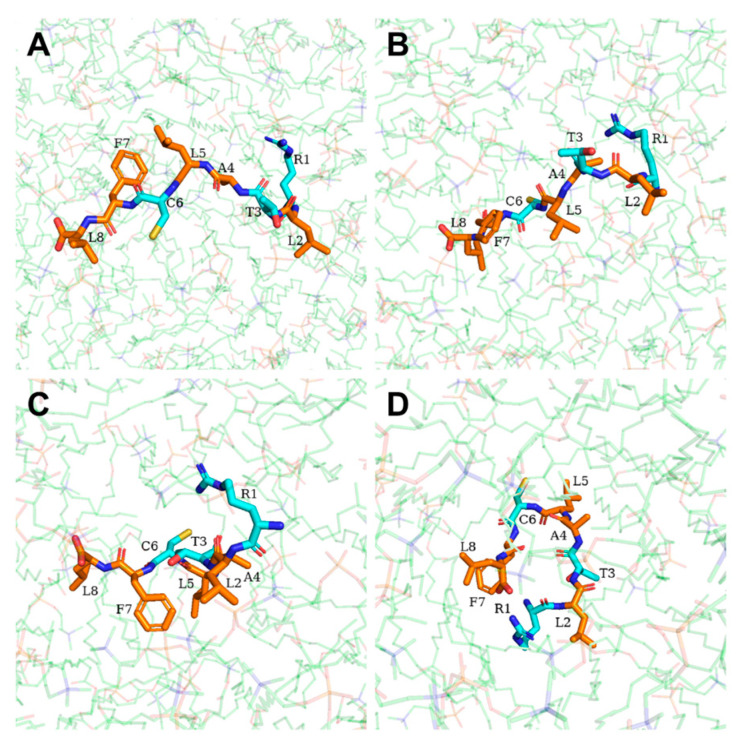
Schematic illustration of DP1 translocation process at (**A**) 200 ps; (**B**) 600 ps; (**C**) 900 ps; (**D**) 1300 ps. Non-polar amino acids are shown in orange. Polar amino acids are shown in cyan. The phospholipid molecules are shown in green.

Our findings suggest that DP1 penetrates the membrane (Video S1) via a mechanism involving several steps that is similar to the barrel-stave mechanism. At 0 ps, DP1 vertically stood in the water molecule layer, with the N-terminus close to the membrane surface (Figure 9A). At 200 ps, the N-terminus of DP1 was bound to the hydrophilic head regions of the upper membrane phospholipids, whereas the C-terminus and the hydrophobic amino acids in the body lay flat on the surface of the upper membrane, forming channels on the membrane surface through hydrophobic interactions (Figure 9B). From 600 ps to 900 ps, the C-terminus of DP1 was pulled along the channel to enter the hydrophobic regions of the membrane completely and moved towards the lower membrane, while the N-terminus was attracted by the hydrophilic head region, resulting in the rotation of the body (Figure 9C,D). At 1300 ps, DP1 was in the shape of a ring close to the lower membrane, with the N-terminus probing from the channel (Figure 9E). At 1500 ps, DP1 penetrated the lower membrane into the water molecule layer (Figure 9F). Throughout transmembrane penetration, polar amino acids (R1, T3, and C6) consistently engage with water molecules and the hydrophilic heads of phospholipids. As the penetration proceeds, they persistently interact with the hydrophilic head regions while evading the hydrophobic tail regions (Figure 10).

**Figure 11:**
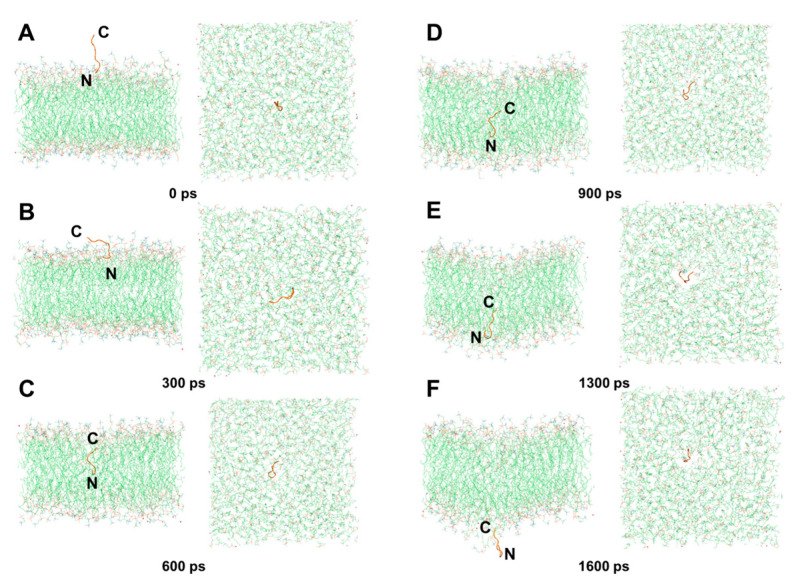
Translocation process diagram of DP1, with the N-terminus approaching the surface of the cell membrane. (**A**) 0 ps. (**B**) 300 ps. (**C**) 600 ps. (**D**) 900 ps. (**E**) 1300 ps. (**F**) 1600 ps.

**Figure 12:**
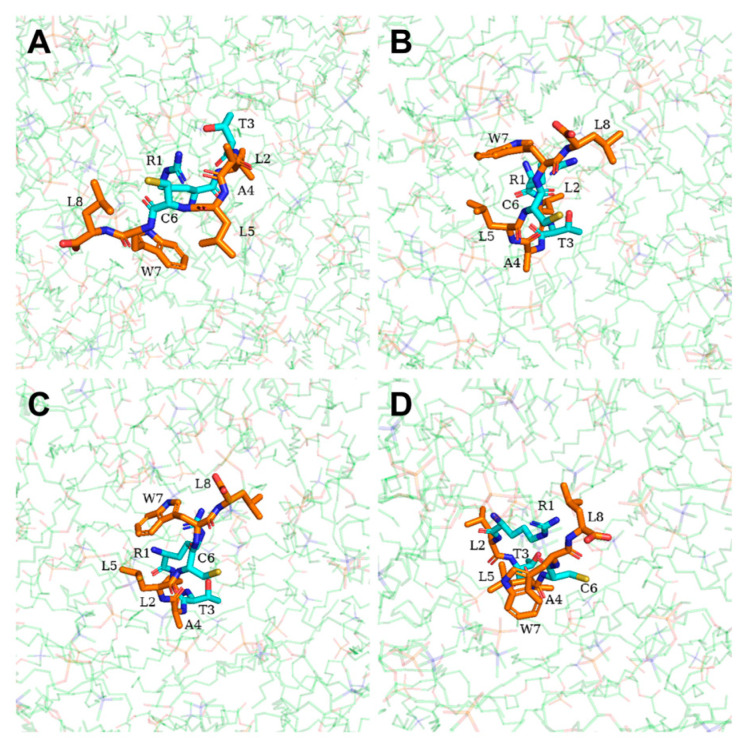
Schematic illustration of the DP2 translocation process at (**A**) 300 ps; (**B**) 600 ps; (**C**) 900 ps; (**D**) 1300 ps. Non-polar amino acids are shown in orange. Polar amino acids are shown in cyan. The phospholipid molecules are shown in green.

Interestingly, we found that the mechanism of DP2 penetration through the membrane (Video S2) was similar to the barrel-stave mechanism as well. At 0 ps, DP2 was perpendicular to the water molecule layer, its N-terminus near the membrane surface (Figure 11A). At 300 ps, DP2’s N-terminus bound to the upper membrane’s hydrophilic head region, while its C-terminus and hydrophobic amino acids formed a channel via surface aggregation (Figure 11B), akin to DP1’s state at 200 ps. From 600 ps to 900 ps, DP2 was pulled by hydrophobic interactions along the channel to completely enter the hydrophobic interior and continuously move to the lower membrane (Figure 11C,D). At 1300 ps, the N-terminus of DP2 arrived at the lower membrane first (Figure 11E), which recapitulates the process at 300 ps. At 1600 ps, DP2 completely penetrated through the lower membrane to enter the water molecule layer (Figure 11F). Similarly, polar amino acids (R1, T3, and C6) consistently engaged with water molecules and the phospholipid’s hydrophilic head, interacting while avoiding the hydrophobic tail region (Figure 12).

From the above dynamic analysis, both DP1 and DP2 reflect a transmembrane mechanism similar to the barrel-stave model, with DP1 traversing the membrane slightly faster than DP2. This difference arises partly due to their amino acid disparity at position 7, where PHE (F) for DP1 and TRP (W) for DP2, both belonging to aromatic amino acids, share a similar aromatic ring structure. This structural similarity translates into functional similarity. Additionally, DP1’s higher overall hydrophobicity compared to DP2 further facilitates membrane penetration. It also emphasizes the importance of hydrophobic amino acids in the process of antimicrobial peptides crossing the cell membrane.

### 2.8. Prediction of Antimicrobial Activity and Toxicity

We chose NoPv1, DP1, and DP2 to predict the antimicrobial activity, and the results are presented in Table 6. The AI4AMP output includes AMP scores ranging from 0 to 1 and prediction results (either “yes” or “no”) for each input sequence. The score indicates the tendency towards being an antimicrobial peptide (AMP), while the prediction result is determined based on a threshold of 0.5. Peptides with elevated AMP scores are likelier to exhibit substantial antimicrobial efficacy. It showed that both NoPv1 and its mutants are antimicrobial peptides without being toxic for human cells, while the mutant DP1 holds a more remarkable antimicrobial effect due to its highest predicted score.

## 3. Materials and Methods

### 3.1. Preparation of Protein Receptors and Ligands

The amino acid sequence of the protein PvCesA2 (UniProtKB: D4N2S6, from aa 331 to 790) was obtained from the UniProt database (https://www.uniprot.org/, accessed on 12 October 2022) and a series of NoPv peptides were obtained from the established library of eight amino acid peptides [24]. Their 3D crystal structure was predicted using AlphaFold2 (ColabFold: AlphaFold2 using MMseqs2) [25]. The AlphaFold2 network predicts the 3D coordinates of all heavy atoms for a given protein using its primary amino acid sequence [26]. The AlphaFold2 algorithm framework utilizes multiple sequence alignment (MSA) and amino acid pairs (pairwise) of proteins in a collaborative learning approach. It integrates evolutionary information on protein sequences as well as physical and geometric constraints on protein structures into deep learning networks. The quality of the resulting model is assessed using parameters such as the predicted local distance difference test (pLDDT), which is scored on a scale from 0 to 100. The 3D crystal structure of PvCesA2 and NoPv peptides was obtained by comparing pLDDT and pre-processed using DISCOVERY STUDIO v4.5 software, where all water molecules were removed, incomplete amino acid side chains or missing atoms from the main chain and hydrogen atoms were added.

### 3.2. Molecular Docking (ZDOCK)

The protein-protein docking simulations were performed using the DISCOVERY STUDIO v4.5 in Dock Proteins (ZDOCK) protocol, which employs rigid body docking of two protein structures utilizing the ZDOCK algorithm. Additionally, it clusters the poses based on the ligand position [27]. ZDOCK is a rigid-body docking program designed for initial-stage unbound docking, requiring minimal information about the binding site. It utilizes a simple shape complementarity method known as Pairwise Shape Complementarity (PSC). Optionally, the PSC method can be enhanced with desolvation (DE) and electrostatic (ELEC) energy terms to rank the docked poses. Unlike explicitly factoring in protein surface curvature or area, PSC values all nearby atomic contacts between the protein receptor and ligand within a defined cutoff distance. The Z-dock score represents the shape complementarity score computed by the ZDOCK program, utilized for assessing the optimal pose [28]. The docking simulations generated 3600 poses, from which 2000 superior poses were selected and then grouped into 60 clusters based on a root-mean-square deviation (RMSD) threshold of 0.6.

Given the Z-dock scores and the structures, we chose the most suitable poses of complexes (PvCesA2-NoPv1, PvCesA2-NoPv2, PvCesA2-NoPv3, PvCesA2-NoPv1R1A, PvCesA2-NoPv1R1AR7A, and PvCesA2-NoPv1R7A), with the Z-dock scores found to be 13.98, 13.5, 12.28, 13.32, 13.16, and 12.7, respectively. The binding energy and the equilibrium dissociation constant (K_D_) were calculated by the YASARA software, version 19.5.23. The most stable conformation of the receptor was determined based on the ligand with the lowest binding energy, indicated by the most negative value [29]. The tighter the ligand-protein binding, or the greater the affinity between them, the smaller the dissociation constant [30].

### 3.3. Calculate Mutation Energy (Binding)

To obtain peptides (based on NoPv peptides) that can bind more strongly to PvCesA2, the single- and double-point saturation mutagenesis of the 8 amino acids was executed using the Calculate Mutation Energy (Binding) protocol in DISCOVERY STUDIO v4.5. Combinatorial amino acid scanning mutagenesis involves mutating selected amino acid residues to one or more specified amino acid types. All energy terms, including electrostatic energy, are calculated using the CHARMm force field, with the electrostatic energy specifically computed using a Generalized Born implicit solvent model. The energy impact of each mutation on the binding affinity, referred to as the mutation energy (∆∆Gmut) is computed using the CHARMm force field and a pH-dependent mode [31]. The pH value, ionic strength, solvent dielectric constant, and energy cutoff were set at 7.4, 0.1, 80, and 0.5, respectively.

(1) $$∆∆Gmut=∆∆Gbind\left(mutant\right)-∆∆Gbind\left(wild\;type\right)$$

where $∆∆Gbind\left(mutant\right)$ represents the binding free energy in the mutated structure, $∆∆Gbind\left(wild\;type\right)$ represents the binding free energy in the wild-type structure. The mutation energy represents the stability of the complex; in other words, a lower mutation energy denotes a more stable structure of the complex.

### 3.4. Molecular Dynamics Simulation

To validate the protein-protein docking model, molecular dynamics (MD) simulation was conducted using the AMBER14 force field [32] in the YASARA program. Periodic boundary conditions and the particle-mesh Ewald method [33] were employed for treating long-range Coulomb forces beyond a 12 Å cutoff. The MD simulation utilized NaCl at a concentration of 0.9% and an HOH density of 0.997 g/mL in the simulation cell. No restraints were applied during the 300 ns simulation, which saved energies and coordinates every 100 ps. The simulation was carried out at a constant temperature of 298 K in the NVT ensemble with uncontrolled pressure.

### 3.5. Dynamic Simulation of Membrane Penetration Mechanism

The Membrane Builder in CHARMM-GUI (https://charmm-gui.org, accessed on 31 January 2024) [34] was used for building up the peptide-membrane system. The membrane is positioned in the X-Y plane with the *Z*-axis perpendicular to it. We uploaded a peptide PDB file and positioned the peptide 3 nm above the initial membrane layer. The upper and lower membranes were composed entirely of POPC molecules. The water thickness was set to 22.5 Å on the top and bottom of the system. Na^+^ and Cl^−^ ions were added using a Monte Carlo-based method. The system was equilibrated at a temperature of 298 K. The force field was selected as AMBER19SB [35]. Following successful system construction, we initiated energy minimization, followed by six rounds of equilibration steps using the GROMACS software (version 2022.4 in the Ubuntu 20.04 environment). After achieving equilibrium, we conducted a 2000-ps molecular dynamics simulation for membrane traversal. The leap-frog algorithm with a time step of 0.002 fs, the particle-mesh Ewald method for electrostatic interactions, and a cutoff for van der Waals interactions were utilized. Bond lengths were constrained via the LINCS algorithm, and an umbrella potential was applied to pull the peptide’s center of mass downward along the *Z*-axis at a rate of 0.005 nm/ps, with a force constant of 1500 kJ/(mol·nm^2^) [36].

### 3.6. Visualization of Protein-Ligand Complexes

The interactions between peptides and PvCesA2 were further studied using the “Ligand Interactions” tool within the “Receptor-Ligand Interactions” module of DISCOVERY STUDIO v4.5 software. This tool characterizes and visualizes weak non-bond forces [37] like hydrogen bonds and hydrophobic contacts, along with distances and dihedral angles, to identify ligand binding sites and amino acid interactions. Furthermore, the 3D molecular interactions of all complexes were visualized in PyMOL version 2.5.4 by Schrödinger [38].

### 3.7. Quantum Chemical Analysis

The selected ligands underwent structural analysis using GAUSSIAN 09 [39], while Multiwfn version 3.8 [40] and VMD [41] software version 1.9.3 were utilized to visualize the orbitals. Optimization of the NoPv peptides was achieved through density functional theory (DFT) using the B3LYP-D3/6-31+G(d,p) level [42,43].

### 3.8. Antimicrobial Activity and Toxicity Prediction

The antimicrobial activity of the NoPv peptides was predicted using the open-access online server AI4AMP (https://axp.iis.sinica.edu.tw/AI4AMP, accessed on 6 March 2023) [44]. Utilizing input files containing balanced AMP and non-AMP data, AI4AMP’s deep learning model training and testing processes, along with the PC6 encoding method, were tailored for insertion into this server via the auto-covariance (AC) method. These files comprise the AMP dataset sourced from four databases: APD3 [12], LAMP [45], CAMP3 [46], and DRAMP [47], whereas the non-AMP dataset consists of a blend of real-world peptides and artificially generated sequences. The AC method incorporates four clusters (hydrophobicity (H1), side chain volume (V), polarity (Pl), and pH at the isoelectric point (pI)), alongside two common physicochemical properties (the dissociation constant for the -COOH group (pKa) and the net charge index of the side chain (NCI)), which reflect AMP’s cationic characteristic [48]. Notably, the valid codes should be within the characters ACDEFGHIKLM-NPQRSTVWY, and AI4AMP can only be used to determine whether an input sequence is an antimicrobial peptide and cannot provide specific information about its resistance to particular pathogens. Either type in sequences in FASTA format or upload a FASTA file to “Fileupload (*.txt).” is available. Then we browse the scores and prediction results on the result page and retrieve the output CSV file in the download area.

The toxicity of the peptides was predicted using the user-friendly online web server ToxIBTL (https://server.wei-group.net/ToxIBTL, accessed on 6 March 2023) [49]. Based on the method of ToxIBTL, a novel deep learning framework was built to predict the toxicity of peptides as well as proteins, consisting of three main steps: encoding, optimization, and classification. The first step involves converting raw sequences into evolutionary profiles by BLOSUM62 (BLOcks Substitution Matrix) and using a hybrid network, CNN_BiGRU, to capture information. The second step combines evolutionary and physicochemical features and optimizes them based on the information bottleneck principle and migration learning. Finally, the optimized features are used to determine if the sequence is toxic or non-toxic. After uploading the selected peptide files (in .FASTA format), press the “Submit” button to begin the classification. Waiting a moment, and the results will be presented on the web page.

## 4. Conclusions

Based on a series of computer-aided analysis methods, we verified that NoPv1 has a good inhibitory effect on PvCesA2 compared with other NoPv peptides from the perspective of molecular interaction and dynamic simulation, which is consistent with the known biological activity experimental data, and further concluded the feasibility of NoPv1 as an antimicrobial peptide in the prevention of potato late blight. Based on these findings, we carried out saturation mutations to further improve the antimicrobial activity of NoPv1. According to the results, 9 candidate peptides were selected, and further results revealed that the double mutants at positions Q5 and R7, DP1 and DP2, have higher affinity with PvCesA2, and DP1 in particular exhibited higher antimicrobial activity than NoPv1. Neither DP1 or DP2 were toxic to human cells. Meanwhile, our findings suggest that the transmembrane processes of DP1 and DP2 likely operate via a barrel-stave model, in which the peptide’s hydrophobic amino acids interact with the hydrophobic region of the membrane to open channels on the membrane surface. In addition, the amino acids GLN-326 and TRP-439 on the binding interface of PvCesA2 seem to play indispensable roles in the interaction mechanism between PvCesA2 and NoPv1 with its mutants. Above all, the peptide NoPv1 and its mutants could be potential pesticide candidates for effectively controlling potato late blight by inhibiting the target protein. Based on this computationally derived mechanism, we anticipate that our study may offer insights for advancing the development of eco-friendly pesticides targeting various oomycete diseases, including late blight.

## Figures and Tables

**Table 1:** PvCesA2 models and corresponding pLDDT scores.

Rank	pLDDT
1	88.1
2	87.9
3	86.5
4	80.9
5	79.2

**Table 2:** Partial parameters of NoPv peptides and the corresponding highest pLDDT scores.

Peptides	Amino Acid Sequence	pLDDT	GRAVY ^1^	Charge ^1^
NoPv1	NH_2_-RLTAQCRL-COOH	70.5	−0.163	+2
NoPv2	NH_2_-LFPFVSSM-COOH	70.1	1.538	0
NoPv3	NH_2_-MLLHSELC-COOH	72.8	1.038	0
NoPv1R1A	NH_2_-ALTAQCRL-COOH	71.7	0.625	+1
NoPv1R1AR7A	NH_2_-ALTAQCAL-COOH	68.0	1.413	0
NoPv1R7A	NH_2_-RLTAQCAL-COOH	67.5	0.625	+1

^1^ GRAVY and Charge were calculated from the APD3 website [12].

**Table 3:** Results of the molecular docking between the NoPv peptides and PvCesA2.

Ligand	Receptor	Binding Free Energies [kcal/mol]	K_D_ [μM]
NoPv1	PvCesA2	−6.949	8.060
NoPv2		−6.334	22.76
NoPv3		−4.744	333.1
NoPv1R1A		−6.567	15.36
NoPv1R1AR7A		−6.268	25.44
NoPv1R7A		−6.310	23.70

**Table 4:** The binding affinity of the native and mutated peptides with PvCesA2.

Peptides	Amino Acid Sequence	Binding Free Energies [kcal/mol]
NoPv1	NH_2_-RLTAQCRL-COOH	−6.949
SP1 R1P	NH_2_-PLTAQCRL-COOH	−6.434
SP2 L2P	NH_2_-RPTAQCRL-COOH	−6.758
SP3 T3A	NH_2_-RLAAQCRL-COOH	−6.539
SP4 A4M	NH_2_-RLTMQCRL-COOH	−6.525
SP5 Q5L	NH_2_-RLTALCRL-COOH	−7.152
SP6 C6H	NH_2_-RLTAQHRL-COOH	−7.139
SP7 R7F	NH_2_-RLTAQCFL-COOH	−7.383
DP1 Q5L R7F	NH_2_-RLTALCFL-COOH	−7.564
DP2 Q5L R7W	NH_2_-RLTALCWL-COOH	−7.551

**Table 5:** FMOs results for the three ligands.

Ligand	HOMO (eV)	LUMO (eV)	∆*E*_*L*-*H*_ = E_LUMO_ − E_HOMO_ (eV)
NoPv1	−0.164	−0.032	0.132
DP1	−0.227	−0.048	0.179
DP2	−0.210	−0.047	0.163

**Table 6:** Prediction results of the peptides.

Peptides	AMP Score	AMP Result	Toxicity
NoPv1	0.82	yes	no
DP1	0.90	yes	no
DP2	0.52	yes	no

## Data Availability

All the data are included in this paper.

## Supplementary Materials

The following supporting information can be downloaded at: https://www.mdpi.com/article/10.3390/ijms25105312/s1.
