# Supplementary material for: Mechanism Analysis of Antimicrobial Peptide NoPv1 Related to Potato Late Blight through a Computer-Aided Study
Source: Int J Mol Sci. 2024 May 13;25(10):5312. doi: 10.3390/ijms25105312 (PMC11121460; doi:10.3390/ijms25105312)
Supplement: Supplementary file 1 [file ijms-25-05312-s001.zip › supporting information.pdf]

Supporting Information

Mechanism analysis of antimicrobial peptide NoPv1 related to potato late blight through a computer-aided study

Jiao-shuai Zhou [1,2], Hong-liang Wen [1,2,*] and Ming-jia Yu [1,*]

[1] School of Chemistry and Chemical Engineering, Beijing Institute of Technology, Beijing 100081, China; zjs2021@bit.edu.cn (J.-S.Z.)

[2] Key Laboratory of Medical Molecule Science and Pharmaceutical Engineering, Yangtze Delta Region Academy of Beijing Institute of Technology, Jiaxing 314019, China.

[*] Correspondence: wen.hongliang@bit.edu.cn (W.H.-L.); 6120210204@bit.edu.cn (Y.M.-J.)

Contents:

Table S1. The interaction result between PvCesA2 and NoPv1 obtained from docking.

Table S2. The interaction result between PvCesA2 and NoPv1R1A obtained from docking.

Table S3. The interaction result between PvCesA2 and NoPv1R1AR7A obtained from docking.

Table S4. The interaction result between PvCesA2 and NoPv1R7A obtained from docking.

Table S5. The interaction result between PvCesA2 and NoPv2 obtained from docking.

Table S6. The interaction result between PvCesA2 and NoPv3 obtained from docking.

Table S7. The interaction result between PvCesA2 and DP1 obtained from docking.

Table S8. The interaction result between PvCesA2 and DP2 obtained from docking.

Table S9. The detailed interaction result of the PvCesA2-NoPv1 complex conformations every 30 ns during the MD simulation (300 ns).

Table S10. The detailed interaction result of the PvCesA2-DP1 complex conformations every 30 ns during the MD simulation (300 ns).

Table S11. The detailed interaction result of the PvCesA2-DP2 complex conformations every 30 ns during the MD simulation (300 ns).

Figure S1. ZDOCK scores of top ranked poses and the chosen one. A. PvCesA2-NoPv1 B. PvCesA2-NoPv2. C. PvCesA2-NoPv3. D. PvCesA2-NoPv1R1A. E. PvCesA2-NoPv1R1AR7A. F. PvCesA2-NoPv1R7A.

Figure S2. Predicted combined mutation energy of PvCesA2-NoPv1 binding at pH 7.4.

Figure S3. Interaction of mutants of NoPv1 with PvCesA2 after saturation mutations. A. Interaction between DP1 and PvCesA2. B. Interaction between DP2 and PvCesA2. The green dashed lines represent conventional hydrogen bonds.

Figure S4. The RMSD profiles of 300 ns MD simulations of complexes and protein. A. PvCesA2 and NoPv1. B. PvCesA2 and DP1. C. PvCesA2 and DP2. D. PvCesA2. The RMSD values of Calpha [RMSDCa], backbone [RMSDBb], and all heavy atoms [RMSDAll] are shown in red, blue, and green, respectively.

Figure S5. The relevant analysis of 300 ns MD simulations of complexes and protein. A. The plot of radius of gyration of complexes and protein at 300 ns simulation time. B. The RMSF values of complexes and protein at 300 ns simulation. The parameters of NoPv1, DP1, DP2, and protein are shown in red, blue, green, and purple, respectively.

Figure S6. The Binding Energy profiles of 300 ns MD simulations of PvCesA2 with NoPv1/ DP1/DP2. The Binding Energy values of NoPv1, DP1, and DP2 are shown in red, blue, and green, respectively.

Figure S7. Superposition of two complexes including the last structures obtained from the MD and the complexes obtained from docking. The RMSD values of the structures aligned by PyMOL including the last structure from the MD (colored in light teal) and the complexes obtained from docking (colored in pale green) are 2.015, 1.867, and 1.981, respectively. A. PvCesA2-NoPv1. B. PvCesA2-DP1. C. PvCesA2-DP2.

Figure S8. Interactions of the final structures obtained from the MD. A. Interaction between NoPv1 and PvCesA2. B. Interaction between DP1 and PvCesA2. C. Interaction between DP2 and PvCesA2. The green dashed lines represent conventional hydrogen bonds.

Table S1. The interaction result between PvCesA2 and NoPv1 obtained from docking.

Atoms in the Residues of PvCesA2	Atoms in the Residues of NoPv1	Hydrogen Bond [Å]	Hydrophobic Contact [Å]
HIS157/HN	LEU8/OXT	1.6600	/
TYR158/HN	ARG7/O	2.5647	/
SER159/HN	ARG7/O	2.3475	/
LYS160/HN	GLN5/O	2.4701	/
GLU388/HN	ALA4/O	2.6063	/
GLN326/OE1	ARG1/HH11	1.8023	/
GLN435/OE1	ARG1/HH21	2.8167	/
LEU424/O	ARG1/HH22	2.6976	/
ASP389/OD2	ALA4/HN	1.7818	/
LEU386/O	CYS6/HN	2.7937	/
THR156/HA	LEU8/OXT	2.4569	/
SER159/HB1	GLN5/O	2.4497	/
ARG438/HD1	THR3/OG1	2.3431	/
TRP439/HA	LEU2/O	2.0402	/
GLN326/OE1	ARG1/HD2	2.7219	/
ASP389/OD1	LEU2/HA	2.6985	/
LEU386/O	THR3/HA	2.9977	/
LEU386/O	THR3/HB	2.8268	/
LEU386/O	GLN5/HA	2.8868	/
GLU388/OE2	CYS6/HA	2.8386	/
TRP439	ARG1/LEU2	/	4.2884
TRP439	ARG1/LEU2	/	5.2437
ALA364	LEU2	/	5.3375
CYS390	LEU2	/	4.0776
LYS160	ALA4	/	4.1172
PHE347	LEU2	/	4.9718
TRP439	ARG1	/	4.7773
TRP439	ARG1	/	4.3163

Note: The highlighted indicate amino acids contributing to the interactions between PvCesA2 and each peptide.

Table S2. The interaction result between PvCesA2 and NoPv1R1A obtained from docking.

Atoms in the Residues of PvCesA2	Atoms in the Residues of NoPv1R1A	Hydrogen Bond [Å]	Hydrophobic Contact [Å]
LYS160/HN	ALA4/O	2.7396	/
GLY384/HN	ALA1/O	3.0680	/
GLN435/HE22	LEU8/OXT	2.9551	/
ARG438/HH11	ARG7/O	2.6583	/
ARG438/HH12	ARG7/O	2.6739	/
SER385/O	GLN5/HN	2.9042	/
LEU386/O	GLN5/HN	3.0345	/
GLU388/OE2	CYS6/HN	2.3205	/
HIS21/O	ARG7/HH22	2.3923	/
SER159/HB1	ALA4/O	2.6735	/
GLY384/HA1	ALA1/O	2.255	/
ARG438/HD2	GLN5/O	2.2187	/
SER385/O	ALA4/HA	2.1216	/
ALA161	ALA4	/	3.6043
ALA364	LEU8	/	5.1014
ARG438	CYS6	/	4.3308
LYS160	ARG7	/	4.6956
TYR158	ALA1	/	5.4464
TRP439	LEU8	/	4.9738
TRP439	LEU8	/	5.2676

Note: The highlighted indicate amino acids contributing to the interactions between PvCesA2 and each peptide.

Table S3. The interaction result between PvCesA2 and NoPv1R1AR7A obtained from docking.

Atoms in the Residues of PvCesA2	Atoms in the Residues of NoPv1R1AR7A	Hydrogen Bond [Å]	Hydrophobic Contact [Å]
LYS160/HN	ALA4/O	2.4519	/
GLY384/HN	LEU2/O	2.4415	
ARG438/HH11	GLN5/O	2.5423	
TRP439/HE1	ALA7/O	1.9781	
GLU388/OE1	CYS6/HN	2.7098	
ASP182/OD2	CYS6/HG	2.5478	
ARG438/HD1	GLN5/O	2.3568	
GLU388/OE2	ALA4/HA	2.0533	
ALA161	ALA4	/	3.4848
ALA364	LEU8	/	4.9942
LYS160	ALA4	/	4.8927
HIS157	ALA1	/	4.1129
TRP439	ALA7	/	5.2446
TRP439	LEU8	/	4.9456
TRP439	LEU8	/	4.4684

Note: The highlighted indicate amino acids contributing to the interactions between PvCesA2 and each peptide.

Table S4. The interaction result between PvCesA2 and NoPv1R7A obtained from docking.

Atoms in the Residues of PvCesA2	Atoms in the Residues of NoPv1R7A	Hydrogen Bond [Å]	Hydrophobic Contact [Å]
SER228/HN	GLN5/OE1	2.2650	/
SER228/HG	ARG1/O	2.0414	/
MET266/O	THR3/HG1	2.2274	/
ASP275/O	GLN5/HE22	2.5913	/
ALA271/O	ALA7/HN	2.3396	/
SER228/HA	ARG1/O	2.8381	/
SER228/HB1	GLN5/OE1	2.5617	/
SER228/HB2	ARG1/O	2.7801	/
HIS258/HE1	GLN5/OE1	2.7318	/
ASN263/HA	LEU2/O	2.8845	/
ALA271/HA	THR3/O	1.8964	/
ASP268/O	THR3/HB	2.4908	/
ASN270/O	ALA4/HA	1.9381	/
LYS273/O	GLN5/HA	2.8936	/
VAL253/O	CYS6/HA	2.0492	/
ILE231	LEU2	/	5.2290
ALA232	LEU2	/	5.1571
ARG259	CYS6	/	4.1977
VAL262	LEU2	/	4.9779
MET266	LEU2	/	5.3679
ILE274	LEU8	/	5.4242
ILE231	ARG1	/	5.1106
LYS273	ALA4	/	4.6304

Note: The highlighted indicate amino acids contributing to the interactions between PvCesA2 and each peptide.

Table S5. The interaction result between PvCesA2 and NoPv2 obtained from docking.

Atoms in the Residues of PvCesA2	Atoms in the Residues of NoPv2	Hydrogen Bond [Å]	Hydrophobic Contact [Å]
SER228/HG	PHE2/O	1.9155	/
SER272/HN	VAL5/O	2.8182	/
LYS273/HN	VAL5/O	2.2323	/
MET266/SD	LEU1/HT1	2.6685	/
SER228/HA	PHE2/O	2.9837	/
VAL253/HA	SER6/O	2.9316	/
VAL262/HA	PRO3/O	2.8649	/
SER272/HA	MET8/O	2.6311	/
ASN270/O	VAL5/HA	2.2204	/
LYS273/O	SER6/HN	2.6341	/
SER228/OG	SER6/HB1	2.7043	/
LYS273/O	SER6/HB1	2.8633	/
VAL253/O	SER7/HA	2.5141	/
ASN263/OD1	SER7/HB2	2.9362	/
VAL262	PRO3	/	5.4399
ALA271	MET8	/	5.0553
LYS273	VAL5	/	4.8765
ILE231	PRO3	/	4.7285

Note: The highlighted indicate amino acids contributing to the interactions between PvCesA2 and each peptide.

Table S6. The interaction result between PvCesA2 and NoPv3 obtained from docking.

Atoms in the Residues of PvCesA2	Atoms in the Residues of NoPv3	Hydrogen Bond [Å]	Hydrophobic Contact [Å]
THR265/HN	SER5/OG	2.4757	/
MET266/HN	SER5/OG	2.2896	/
LYS273/HN	GLU6/OE1	2.7088	/
SER228/HA	LEU7/O	2.4455	/
HIS258/HD2	HIS4/NE2	2.6282	/
ASN263/HA	LEU3/O	2.4903	/
ASN263/OD1	HIS4/HA	2.478	/
ASN263/OD1	HIS4/HD2	1.8769	/
MET266/O	SER5/HA	2.3696	/
ASN263/O	SER5/HB1	3.0287	/
MET266/O	SER5/HB1	2.6264	/
VAL262/O	SER5/HB2	2.1838	/
ILE231	LEU7	/	5.2966
ALA232	LEU7	/	4.7303
ALA271	LEU2	/	5.2960
ILE231	CYS8	/	5.4639
ALA271	HIS4	/	4.7697

Note: The highlighted indicate amino acids contributing to the interactions between PvCesA2 and each peptide.

Table S7. The interaction result between PvCesA2 and DP1.

Atoms in the Residues of PvCesA2	Atoms in the Residues of DP1	Hydrogen Bond [Å]	Hydrophobic Contact [Å]
HIS157/HN	LEU8/OXT	1.6606	/
TYR158/HN	PHE7/O	2.5647	/
SER159/HN	PHE7/O	2.3475	/
LYS160/HN	LEU5/O	2.4701	/
GLU388/HN	ALA4/O	2.6063	/
GLN326/OE1	ARG1/HH11	1.8023	/
GLN435/OE1	ARG1/HH21	2.8168	/
LEU424/O	ARG1/HH22	2.6976	/
ASP389/OD2	ALA4/HN	1.7818	/
LEU386/O	CYS6/HN	2.7937	/
THR156/HA	LEU8/OXT	2.4594	/
SER159/HB1	LEU5/O	2.4448	/
TRP439/HA	LEU2/O	2.0402	/
GLN326/OE1	ARG1/HD2	2.7219	/
ASP389/OD1	LEU2/HA	2.6985	/
LEU386/O	THR3/HA	2.9977	/
LEU386/O	THR3/HB	2.8268	/
LEU386/O	LEU5/HA	2.8868	/
TRP439	ARG1/LEU2	/	4.2884
TRP439	ARG1/LEU2	/	5.2437
ALA364	LEU2	/	5.3375
CYS390	LEU2	/	4.0776
ARG438	LEU5	/	5.4580
LYS160	ALA4	/	4.1026
PHE347	LEU2	/	4.9718
TRP439	ARG1	/	4.7773
TRP439	ARG1	/	4.3163

Table S8. The interaction result between PvCesA2 and DP2.

Atoms in the Residues of PvCesA2	Atoms in the Residues of DP2	Hydrogen Bond [Å]	Hydrophobic Contact [Å]
HIS157/HN	LEU8/OXT	1.6606	/
TYR158/HN	TRP7/O	2.5647	/
SER159/HN	TRP7/O	2.3475	/
LYS160/HN	LEU5/O	2.4701	/
GLU388/HN	ALA4/O	2.6063	/
GLN326/OE1	ARG1/HH11	1.8023	/
GLN435/OE1	ARG1/HH21	2.8168	/
LEU424/O	ARG1/HH22	2.6976	/
ASP389/OD2	ALA4/HN	1.7818	/
LEU386/O	CYS6/HN	2.7937	/
THR156/HA	LEU8/OXT	2.4594	/
SER159/HB1	LEU5/O	2.4448	/
TRP439/HA	LEU2/O	2.0402	
GLN326/OE1	ARG1/HD2	2.7219	/
ASP389/OD1	LEU2/HA	2.6985	/
LEU386/O	THR3/HA	2.9977	/
LEU386/O	THR3/HB	2.8268	/
LEU386/O	LEU5/HA	2.8868	/
TRP439	ARG1/LEU2	/	4.2884
TRP439	ARG1/LEU2	/	5.2437
ALA364	LEU2	/	5.3375
CYS390	LEU2	/	4.0776
ARG438	LEU5	/	5.4580
LYS160	ALA4	/	4.1026
PHE347	LEU2	/	4.9718
TRP439	ARG1	/	4.7773
TRP439	ARG1	/	4.3163

Table S9. The detailed interaction result of the PvCesA2-NoPv1 complex conformations every 30 ns during the MD simulation (300 ns).

Time step	Hydrogen Bond<br>Receptor atom—Ligand atom<br>(bond length)	Hydrophobic Contact<br>Receptor residues<br>(bond length)
0 ns	(1) GLN326:OE1—ARG1:H2 (1.82 Å);	(1) LYS160 (3.98 Å);
	(2) TYR348:OH—ARG1:H2 (2.58 Å);	(2) ALA161 (4.97 Å);
	(3) TYR348:OH—ARG1:H3 (2.28 Å);	(3) ALA364 (4.76 Å);
	(4) LEU424:O—ARG1:HH21 (1.89 Å);	(4) HIS157 (4.78 Å);
	(5) GLU388:OE2—CYS6:HG (1.92 Å);	(5) PHE347 (4.66 Å);
	(6) HIS157:H—LEU8:OXT (2.61 Å);	(6) TRP439 (5.41 Å);
	(7) TYR158:H—ARG7:O (2.28 Å);	(7) TRP439 (5.35 Å);
	(8) SER159:H—ARG7:O (2.80 Å);	(8) TRP439 (5.26 Å);
	(9) LYS160:H—GLN5:O (1.91 Å);	
	(10) TYR348:HH—ARG1:O (2.60 Å);	
	(11) ASP389:OD2—THR3:HA (2.55 Å);	
	(12) THR156:HA—LEU8:OXT (2.48 Å);	
	(13) HIS157:HD2—LEU8:OXT (2.39 Å);	
	(14) TYR158:HA—ARG7:O (2.74 Å);	
	(15) SER159:HB1—GLN5:O (2.41 Å);	
	(16) SER159:HB2—GLN5:O (2.65 Å);	
	(17) TRP439:HA—LEU2:O (2.81 Å);	
	(18) TRP439:HD1—LEU2:O (2.88 Å);	
30 ns	(1) HIS342:O—ARG1:H1 (2.36 Å);	(1) LYS160 (4.04 Å);
	(2) GLU326:OE1—ARG1:H2 (1.95 Å);	(2) ALA364 (5.23 Å);
	(3) HIS342:O—ARG1:H3 (2.73 Å);	(3) LEU386 (5.14 Å);
	(4) ASN344:OD1—ARG1:H3 (1.80 Å);	(4) PHE347 (5.20 Å);
	(5) LEU424:O—ARG1:HH21 (1.82 Å);	(5) TRP439 (5.10 Å);
	(6) LEU386:O—THR3:HG1 (1.95 Å);	(6) TRP439 (4.55 Å);
	(7) LEU386:O—ALA4:H (1.99 Å);	
	(8) GLN435:HE21—GLN5:OE1 (1.85 Å);	
	(9) ASP182:OD2—ARG1:HD1 (2.89 Å);	
	(10) ASP182:OD2—ARG1:HD2 (3.01 Å);	
	(11) LEU386:O—THR3:HA (2.81 Å);	
	(12) SER159:HB1—GLN5:O (2.51 Å);	
60 ns	(1) GLN326:OE1—ARG1:H1 (1.91 Å);	(1) TRP439 (2.71 Å);
	(2) ASN344:OD1—ARG1:H2 (1.94 Å);	(2) TYR22 (5.38 Å);
	(3) LEU424:O—ARG1:HH21 (2.03 Å);	(3) ALA364 (5.01 Å);
	(4) SER159:HG—GLN5:O (2.39 Å);	(4) HIS157 (4.00 Å);
	(5) GLN326:HE21—ARG1:O (2.53 Å);	(5) PHE347 (5.00 Å);
	(6) ARG438:HE—THR3:OG1 (2.30 Å);	(6) TRP439 (5.24 Å);
	(7) ARG438:HH21—ALA4:O (2.12 Å);	

	(8) ASP182:OD1—ARG1:HD2 (2.62 Å);	
90 ns	(1) GLN326:OE1—ARG1:H2 (1.78 Å);<br>(2) ASN344:OD1—ARG1:H3 (2.03 Å);<br>(3) TYR22:OH—LEU8:H (2.21 Å);<br>(4) ARG438:HE—THR3:OG1 (1.96 Å);<br>(5) ARG438:HH21—ALA4:O (2.02 Å);<br>(6) ASN344:OD1—ARG1:HA (2.75 Å);<br>(7) ASP182:OD1—ARG1:HD1 (2.67 Å);<br>(8) TYR22:OH—ARG7:HA (2.51 Å);<br>(9) TRP439:HD1—LEU2:O (2.70 Å);	(1) ILE75 (5.10 Å);<br>(2) TYR22 (4.89 Å);<br>(3) TRP439 (4.89 Å);
120 ns	(1) GLN326:OE1—ARG1:H3 (1.84 Å);<br>(2) ASN344:OD1—ARG1:H1 (1.78 Å);<br>(3) ASN344:OD1—LEU2:H (2.14 Å);<br>(4) SER159:O—ARG7:HE (1.81 Å);<br>(5) SER159:O—ARG7:HH21 (2.48 Å);<br>(6) SER159:HG—GLN5:O (1.73 Å);<br>(7) SER159:HB1—GLN5:O (2.87 Å);	(1) TYR22 (4.75 Å);<br>(2) HIS157 (4.81 Å);<br>(3) TRP439 (4.91 Å);
150 ns	(1) GLN326:OE1—ARG1:H1 (2.03 Å);<br>(2) ALA364:O—ARG1:H2 (1.96 Å);<br>(3) GLN435:OE1—THR3:HG1 (1.64 Å);<br>(4) SER159:O—ARG7:HE (1.99 Å);<br>(5) SER159:O—ARG7:HH21 (2.26 Å);<br>(6) TYR22:HH—ARG7:O (1.87 Å);<br>(7) SER159:HG—GLN5:O (1.73 Å);<br>(8) GLN435:HE21—THR3:O (1.88 Å);<br>(9) ARG438:HE—ALA4:O (2.01 Å);<br>(10) ARG438:HH21—ALA4:O (2.39 Å);<br>(11) TRP439:HE1—THR3:OG1 (2.80 Å);<br>(12) TRP439:HD1—THR3:OG1 (2.45 Å);	(1) TRP439 (2.83 Å);<br>(2) TYR22 (4.65 Å);<br>(3) HIS157 (5.17 Å);<br>(4) TRP439 (4.82 Å);
180 ns	(1) GLN326:OE1—ARG1:H1 (1.76 Å);<br>(2) GLN435:OE1—THR3:HG1 (2.20 Å);<br>(3) SER159: O—ARG7:HE (3.02 Å);<br>(4) SER159:O—ARG7:HE21 (2.09 Å);<br>(5) GLN435:HE21—ALA4:O (1.89 Å);<br>(6) GLN435:HE22—GLN5:OE1 (1.74 Å);<br>(7) ARG438:HH12—ALA4:O (2.30 Å);<br>(8) TRP439:HE1—THR3:OG1 (1.82 Å);<br>(9) ASP182:OD1—ARG1:HD1 (2.62 Å);<br>(10) ASP182:OD1—ARG1:HD2 (2.94 Å);	(1) TRP439 (2.77 Å);<br>(2) LYS160 (4.26 Å);<br>(3) TYR22 (4.95 Å);<br>(4) HIS157 (4.48 Å);<br>(5) TRP439 (4.85 Å);

210 ns	(1) GLN326:OE1—ARG1:H1 (1.74 Å);<br>(2) ALA434:O—CYS6:HG (2.48 Å);<br>(3) SER159:O—ARG7:HE (2.35 Å);<br>(4) SER159:O—ARG7:HH21 (2.25 Å);<br>(5) TRP439:HE1—THR3:OG1 (1.81 Å);<br>(6) ASP182:OD2—ARG1:HD1 (2.55 Å);<br>(7) TYR22:OH—ARG7:HA (2.31 Å);	(1) LYS160 (4.50 Å);<br>(2) ARG438 (4.57 Å);<br>(3) ALA434 (4.05 Å);<br>(4) TYR22 (4.80 Å);<br>(5) HIS157 (4.94 Å);<br>(6) TRP439 (4.89 Å);
240 ns	(1) GLN326:OE1—ARG1:H1 (1.82 Å);<br>(2) CYS20:SG—ARG7:HH22 (2.97 Å);<br>(3) SER23:HG—CYS6:SG (2.00 Å);<br>(4) TRP439:HE1—THR3:OG1 (2.02 Å);<br>(5) ASP182:OD2—ARG1:HD1 (2.95 Å);	(1) LYS160 (4.33 Å);<br>(2) TYR22 (4.93 Å);
270 ns	(1) GLN326:OE1—ARG1:H2 (2.07 Å);<br>(2) SER159:O—ARG7:HE (2.53 Å);<br>(3) SER23: H—CYS6:SG (2.45 Å);<br>(4) TRP439:HE1—THR3:OG1 (1.83 Å);<br>(5) ASP182:OD2—ARG1:HD1 (2.46 Å);<br>(6) SER159:O—ARG7:HD2 (2.97 Å);<br>(7) TRP439:HD1—THR3:OG1 (3.10 Å);	(1) LYS160 (4.75 Å);<br>(2) TYR22 (4.63 Å);<br>(3) TRP439 (5.49 Å);
300 ns	(1) GLN326:OE1—ARG1:H1 (1.85 Å);<br>(2) ALA364:O—ARG1:H2 (2.03 Å);<br>(3) SER23:HG—CYS6:O (1.92 Å);<br>(4) TRP439:HE1—THR3:OG1 (1.97 Å);<br>(5) ASP182:OD2—ARG1:HD1 (2.33 Å);	(1) PRO25 (5.27 Å);<br>(2) TRP439 (5.18 Å);

Table S10. The detailed interaction result of the PvCesA2-DP1 complex conformations every 30 ns during the MD simulation (300 ns).

Time step	Hydrogen Bond<br>Receptor atom—Ligand atom<br>(bond length)	Hydrophobic Contact<br>Receptor residues<br>(bond length)
0 ns	(1) HIS157:H—LEU8:OXT (2.10 Å);	(1) ALA161 (4.45 Å);
	(2) HIS157: HD1—PHE7:O (1.98 Å);	(2) LYS160 (4.08 Å);
	(3) TYR158:H—PHE7:O (2.60 Å);	(3) CYS390 (5.05 Å);
	(4) SER159:H—PHE7:O (1.96 Å);	(4) TYR158 (4.52 Å);
	(5) LYS160:H—LEU5:O (1.94 Å);	(5) PHE347 (4.42 Å);
	(6) GLN326:OE1—ARG1:H2 (1.75 Å);	(6) TRP439 (5.39 Å);
	(7) TYR348:OH—ARG1:H2 (2.55 Å);	(7) TRP439 (5.31 Å);
	(8) TYR348:OH—ARG1:H3 (2.34 Å);	(8) TRP439 (4.89 Å);
	(9) LEU424:O—ARG1:HH21 (2.09 Å);	
	(10) LEU386:O—THR3:HG1 (1.83 Å);	
	(11) ASP389:OD2—ALA4:H (2.24 Å);	
	(12) THR156:HA—LEU8:OXT (2.43 Å);	
	(13) HIS157:HE1—LEU8:O (2.97 Å);	
	(14) SER159:HB1—LEU5:O (2.46 Å);	
	(15) TRP439:HA—LEU2:O (2.49 Å);	
	(16) TRP439:HA—THR3:OG1 (2.84 Å);	
	(17) ASP389:OD2—THR3:HA (2.67 Å);	
30 ns	(1) GLN435:HE21—THR3:O (2.16 Å);	(1) CYS390 (5.37 Å);
	(2) ALA364:O—ARG1:H1 (1.95 Å);	(2) LEU386 (5.06 Å);
	(3) GLN326:OE1—ARG1:H3 (1.97 Å);	(3) TRP439 (5.47 Å);
	(4) GLN435:OE1—THR3:HG1 (2.27 Å);	
	(5) THR387:HA—LEU2:O (2.69 Å);	
	(6) ARG438:HD1—ALA4:O (2.52 Å);	
	(7) ARG438:HD2—ALA4:O (2.81 Å);	
	(8) TRP439:HD1—THR3:OG1 (2.78 Å);	
	(9) ASP182:OD2—ARG1:HD1 (2.67 Å);	
	(10) ASP182:OD2—ARG1:HD2 (2.50 Å);	
60 ns	(1) SER23:HG—LEU5:O (2.38 Å);	(1) CYS390 (4.92 Å);
	(2) GLN435:HE21—THR3:O (1.84 Å);	(2) PRO426 (5.49 Å);
	(3) ALA364:O—ARG1:H1 (2.31 Å);	(3) ALA430 (5.35 Å);
	(4) GLN326:OE1—ARG1:H3 (2.05 Å);	(4) ILE446 (5.31 Å);
	(5) SER23:HB2—LEU5:O (2.90 Å);	(5) TYR22 (4.52 Å);
	(6) ALA364:HA—ARG1:O (2.85 Å);	(6) TYR22 (5.31 Å);
	(7) TRP439:HD1—THR3:OG1 (2.39 Å);	(7) ILE75 (4.29 Å);
	(8) ASP182:OD2—ARG1:HD2 (2.76 Å);	
	(9) GLN435:OE1—LEU5:HA (2.82 Å);	
90 ns	(1) GLN435:HE21—THR3:O (1.72 Å);	(1) TYR22 (4.61 Å);

	(2) ARG438:HH11—ALA4:O (2.34 Å);<br>(3) ARG438:HH22—CYS6:O (2.08 Å);<br>(4) GLN326:OE1—ARG1:H2 (2.62 Å);<br>(5) GLN326:OE1—ARG1:H3 (2.43 Å);<br>(6) ALA364:O—ARG1:H3 (2.09 Å);<br>(7) GLN435:OE1—THR3:HG1 (2.06 Å);<br>(8) TYR22:OH—PHE7:H (2.40 Å);<br>(9) ALA364:HA—ARG1:O (2.80 Å);<br>(10) ARG438:HD1—ALA4:O (2.55 Å);<br>(11) TRP439:HD1—THR3:OG1 (2.49 Å);	(2) CYS390 (4.90 Å);<br>(3) PRO426 (5.14 Å);<br>(4) ALA434 (4.46 Å);<br>(5) TYR22 (4.52 Å);<br>(6) ILE75 (5.29 Å);
120 ns	(1) CYS390:HG—LEU2:O (2.24 Å);<br>(2) GLN435:HE21—THR3:O (2.00 Å);<br>(3) ARG438:HH11—ALA4:O (2.61 Å);<br>(4) ARG438:HH12—CYS6:O (3.08 Å);<br>(5) ARG438:HH22—CYS6:O (2.10 Å);<br>(6) GLN326:OE1—ARG1:H2 (2.71 Å);<br>(7) GLN326:OE1—ARG1:H3 (2.43 Å);<br>(8) LEU424:O—ARG1:HH21 (2.72 Å);<br>(9) LEU424:O—ARG1:HH22 (2.58 Å);<br>(10) GLN435:OE1—THR3:HG1 (2.33 Å);<br>(11) ALA364:HA—ARG1:O (2.92 Å);<br>(12) THR387:HA—LEU2:O (2.31 Å);<br>(13) ARG438:HD1—ALA4:O (2.98 Å);<br>(14) ARG438:HD2—ALA4:O (3.09 Å);<br>(15) TRP439:HD1—THR3:OG1 (2.40 Å);<br>(16) ASP182:OD2—ARG1:HD1 (2.95 Å);<br>(17) LEU386:O—THR3:HA (2.63 Å);<br>(18) LEU386:O—THR3:HB (2.98 Å);<br>(19) GLN435:OE1—LEU5:HA (2.62 Å);	(1) TRP439 (2.93 Å);<br>(2) CYS390 (5.03 Å);<br>(3) PRO426 (4.59 Å);<br>(4) ALA434 (4.39 Å);
150 ns	(1) GLN435:HE21—THR3:O (1.93 Å);<br>(2) ARG438:HH11—ALA4:O (2.71 Å);<br>(3) GLN326:OE1—ARG1:H1 (2.02 Å);<br>(4) GLN326:OE1—ARG1:H3 (2.48 Å);<br>(5) GLN435:OE1—THR3:HG1 (2.53 Å);<br>(6) TYR22:OH—PHE7:H (2.07 Å);<br>(7) ALA364:HA—ARG1:O (2.92 Å);<br>(8) ARG438:HD1—ALA4:O (2.42 Å);<br>(9) TRP439:HD1—THR3:OG1 (2.51 Å);<br>(10) ASP182:OD2—ARG1:HD1 (2.63 Å);<br>(11) ASP182:OD2—ARG1:HD2 (2.79 Å);	(1) CYS390 (5.13 Å);<br>(2) TRP439 (5.36 Å);
180 ns	(1) GLN435:HE21—THR3:O (1.83 Å);	(1) CYS390 (5.18 Å);

	(2) GLN326:OE1—ARG1:H1 (2.13 Å);<br>(3) ALA364:O—ARG1:H2 (2.18 Å);<br>(4) LEU424:O—ARG1:HH21 (2.28 Å);<br>(5) GLN435:OE1—THR3:HG1 (1.92 Å);<br>(6) TYR22:OH—PHE7:H (1.83 Å);<br>(7) ARG438:HD1—ALA4:O (2.57 Å);<br>(8) ARG438:HD2—ALA4:O (2.64 Å);<br>(9) TRP439:HD1—THR3:OG1 (2.88 Å);<br>(10) ASP182:OD2—ARG1:HD1 (2.55 Å);<br>(11) TYR22:OH—CYS6:HA (2.34 Å);	(2) TYR22 (4.20 Å);<br>(3) HIS157 (4.69 Å);
210 ns	(1) GLN435:HE21—THR3:O (1.85 Å);<br>(2) ARG438:HE—CYS6:O (2.74 Å);<br>(3) ARG438:HH11—ALA4:O (2.17 Å);<br>(4) ARG438:HH21—CYS6:O (2.45 Å);<br>(5) ALA364:O—ARG1:H1 (2.21 Å);<br>(6) GLN326:OE1—ARG1:H3 (1.78 Å);<br>(7) LEU424:O—ARG1:HH21 (2.92 Å);<br>(8) GLN435:OE1—THR3:HG1 (2.22 Å);<br>(9) THR387:HA—LEU2:O (2.76 Å);<br>(10) ARG438:HD1—ALA4:O (2.50 Å);<br>(11) ASP182:OD2—ARG1:HD1 (2.66 Å);<br>(12) ASP182:OD2—ARG1:HD2 (3.05 Å);	(1) CYS390 (5.10 Å);<br>(2) PRO426 (5.31 Å);<br>(3) TYR22 (5.21 Å);<br>(4) HIS157 (4.68 Å);<br>(5) TRP439 (4.58 Å);<br>(6) TRP439 (4.50 Å);
240 ns	(1) GLN435:HE21—THR3:O (2.19 Å);<br>(2) ARG438:HH11—ALA4:O (2.48 Å);<br>(3) ARG438:HH12—LEU5:O (2.93 Å);<br>(4) ALA364:O—ARG1:H1 (1.87 Å);<br>(5) GLN326:OE1—ARG1:H3 (2.06 Å);<br>(6) GLN435:OE1—THR3:HG1 (2.27 Å);<br>(7) THR387:HA—LEU2:O (2.76 Å);<br>(8) THR387:HB—LEU2:O (2.80 Å);<br>(9) ARG438:HD1—ALA4:O (2.95 Å);<br>(10) ARG438:HD2—ALA4:O (2.95 Å);<br>(11) ASP182:OD2—ARG1:HD1 (2.76 Å);<br>(12) ASP182:OD2—ARG1:HD2 (3.02 Å);	(1) CYS390 (5.38 Å);<br>(2) ARG438 (5.26 Å);<br>(3) TYR22 (4.97 Å);<br>(4) TRP439 (5.29 Å);<br>(5) ALA434 (5.34 Å);
270 ns	(1) GLN435:HE21—THR3:O (2.26 Å);<br>(2) GLN435:HE21—ALA4:O (2.31 Å);<br>(3) ARG438:HH11—ALA4:O (2.66 Å);<br>(4) ARG438:HH12—LEU5:O (2.84 Å);<br>(5) GLN326:OE1—ARG1:H1 (2.04 Å);<br>(6) ALA364:O—ARG1:H2 (2.13 Å);<br>(7) GLN435:OE1—THR3:HG1 (2.23 Å);	(1) CYS390 (4.88 Å);<br>(2) PRO426 (5.32 Å);<br>(3) HIS157 (5.25 Å);<br>(4) ALA434 (4.61 Å);

	(8) GLY365:HA2—ARG1:O (2.91 Å);<br>(9) THR387:HA—LEU2:O (3.05 Å);<br>(10) TRP439:HD1—THR3:OG1 (2.36 Å);<br>(11) ASP182:OD2—ARG1:HD1 (2.77 Å);<br>(12) ASP182:OD2—ARG1:HD2 (2.41 Å);	
300 ns	(1) GLN435:HE21—THR3:O (1.97 Å);<br>(2) ARG438:HH11—ALA4:O (2.73 Å);<br>(3) GLN326:OE1—ARG1:H1 (2.18 Å);<br>(4) ALA364:O—ARG1:H2 (2.10 Å);<br>(5) GLN435:OE1—THR3:HG1 (2.06 Å);<br>(6) GLY365:HA2—ARG1:O (2.87 Å);<br>(7) ARG438:HD1—ALA4:O (2.64 Å);<br>(8) TRP439:HD1—THR3:OG1 (2.51 Å);<br>(9) ASP182:OD2—ARG1:HD1 (3.01 Å);<br>(10) ASP182:OD2—ARG1:HD2 (2.76 Å);	(1) CYS390 (4.84 Å);<br>(2) LEU386 (5.41 Å);

Table S11. The detailed interaction result of the PvCesA2-DP2 complex conformations every 30 ns during the MD simulation (300 ns).

Time step	Hydrogen Bond<br>Receptor atom—Ligand atom<br>(bond length)	Hydrophobic Contact<br>Receptor residues<br>(bond length)
0 ns	(1) HIS157:H—LEU8:OXT (2.00 Å);	(1) TYR22 (2.70 Å);
	(2) HIS157: HD1—TRP7:O (1.94 Å);	(2) TYR22 (5.10 Å);
	(3) HIS157: HD1—LEU8:OXT (2.73 Å);	(3) ALA161 (4.16 Å);
	(4) TYR158:H—TRP7:O (2.68 Å);	(4) CYS390 (5.38 Å);
	(5) SER159:H—TRP7:O (2.02 Å);	(5) LYS160 (4.56 Å);
	(6) LYS160:H—LEU5:O (1.86 Å);	(6) TYR158 (4.74 Å);
	(7) TYR348: HH—ARG1:O (2.68 Å);	(7) PHE347 (4.71 Å);
	(8) GLN326:OE1—ARG1:H2 (1.76 Å);	(8) TRP439 (5.14 Å);
	(9) TYR348:OH—ARG1:H3 (2.12 Å);	(9) TRP439 (4.85 Å);
	(10) LEU424:O—ARG1:HH21 (1.80 Å);	
	(11) LEU386:O—THR3:HG1 (1.77 Å);	
	(12) ASP389:OD2—ALA4:H (2.31 Å);	
	(13) TYR158: O—CYS6:HG (2.03 Å);	
	(14) THR156:HA—LEU8:OXT (2.50 Å);	
	(15) TYR158:HA—TRP7:O (2.84 Å);	
	(16) SER159:HB1—LEU5:O (2.30 Å);	
	(17) TRP439:HA—LEU2:O (2.45 Å);	
	(18) TRP439:HD1—LEU2:O (2.61 Å);	
	(19) TRP439:HD1—THR3:OG1 (2.41 Å);	
	(20) LEU386:O—THR3:HA (2.73 Å);	
	(21) TRP439 —LEU2:H (2.60 Å); Pi-Donor	
30 ns	(1) THR156:HG1—LEU8:OXT (1.69 Å);	(1) LEU386 (5.06 Å);
	(2) HIS157:H—LEU8:OXT (1.92 Å);	(2) TRP439 (5.43 Å);
	(3) HIS157:HD1—LEU8:OXT (1.80 Å);	
	(4) THR158:H—LEU8:OXT (1.95 Å);	
	(5) SER159:H—TRP7:O (3.03 Å);	
	(6) SER159:HG—TRP7:O (1.71 Å);	
	(7) ALA364:O—ARG1:H2 (2.07 Å);	
	(8) LEU386:O—CYS6:HG (3.05 Å);	
	(9) THR156:HA—LEU8:OXT (2.70 Å);	
	(10) HIS157:HE1—TRP7:O (2.83 Å);	
	(11) SER159:HB1—LEU5:O (2.69 Å);	
	(12) SER159:HB2—LEU5:O (2.72 Å);	
	(13) ASP182:OD2—ARG1:HD2 (2.76 Å);	
60 ns	(1) ARG438:HH21—LEU5:O (2.72 Å);	(1) TYR22 (5.01 Å);
	(2) GLN326:OE1—ARG1:H2 (2.08 Å);	(2) HIS157 (5.22 Å);
	(3) ALA364:O—ARG1:H3 (2.83 Å);	
	(4) ASP182:OD2—ARG1:HD2 (3.08 Å);	

90 ns	(1) ARG438:HE—LEU8:O (2.59 Å);<br>(2) ALA364:O—ARG1:H2 (2.10 Å);<br>(3) SER159:HB1—LEU8:OXT (2.42 Å);<br>(4) GLN326:OE1—ARG1:HA (2.59 Å);<br>(5) ASP182:OD2—ARG1:HD2 (2.44Å);<br>(6) GLN435:OE1—THR3:HA (2.71 Å);	(1) LYS160 (5.17 Å);<br>(2) ALA434 (4.68 Å);<br>(3) TYR22 (4.36 Å);<br>(4) TRP439 (4.71 Å);<br>(5) TRP439 (5.10 Å);
120 ns	(1) GLN435:HE21—ALA4:O (2.68 Å);<br>(2) GLN435:HE21—CYS6:SG (2.58 Å);<br>(3) ALA364:O—ARG1:H2 (2.00 Å);<br>(4) GLN435:OE1—ALA4:H (2.04 Å);<br>(5) GLN326:OE1—ARG1:HA (2.92 Å);<br>(6) ASP182:OD2—ARG1:HD1 (2.68 Å);	(1) ARG436 (5.06 Å);<br>(2) TYR22 (5.49 Å);<br>(3) TRP439 (4.58 Å);<br>(4) TRP439 (5.09 Å);
150 ns	(1) ARG438:HE—LEU8:O (1.83 Å);<br>(2) ALA364:O—ARG1:H3 (1.74 Å);<br>(3) ARG438:HD1—CYS6:O (2.50 Å);<br>(4) ASP182:OD1—ARG1:HD1 (2.74 Å);<br>(5) ASP182:OD2—ARG1:HD2 (2.83 Å);<br>(6) GLN435:OE1—THR3:HA (2.59 Å);	(1) TYR22 (5.72 Å);<br>(2) TRP439 (4.58 Å);<br>(3) ARG436 (4.51 Å);<br>(4) ARG438 (4.62 Å);<br>(5) HIS157 (4.79 Å);<br>(6) TRP439 (4.49 Å);<br>(7) TRP439 (4.90 Å);
180 ns	(1) ARG438:HE—LEU8:OXT (1.93 Å);<br>(2) ALA364:O—ARG1:H2 (1.73 Å);<br>(3) GLN435:OE1—ALA4:H (2.62 Å);<br>(4) GLN326:OE1—ARG1:HA (2.53 Å);<br>(5) ASP182:OD2—ARG1:HD1 (2.91 Å);<br>(6) ASP182:OD2—ARG1:HD2 (2.78 Å);	(1) ALA434 (4.18 Å);<br>(2) ARG436 (4.70 Å);<br>(3) TYR22 (4.44 Å);<br>(4) HIS157 (4.98 Å);<br>(5) TRP439 (4.43 Å);<br>(6) TRP439 (4.42 Å);
210 ns	(1) ARG438:HE—LEU8:O (2.07 Å);<br>(2) ALA364:O—ARG1:H2 (2.15 Å);<br>(3) GLN435:OE1—ALA4:H (2.19 Å);<br>(4) SER159:OG—LEU5:H (2.06 Å);<br>(5) ASP182:OD2—ARG1:HD1 (2.97 Å);<br>(6) ASP182:OD2—ARG1:HD2 (2.86 Å);<br>(7) GLN435:OE1—THR3:HA (2.30 Å);<br>(8) GLN435:OE1—THR3:HB (2.54 Å);	(1) ALA434 (4.75 Å);<br>(2) ARG436 (4.72 Å);<br>(3) ARG438 (5.13 Å);<br>(4) TYR22 (4.84 Å);<br>(5) HIS157 (5.47 Å);<br>(6) HIS157 (5.04 Å);<br>(7) TRP439 (4.92 Å);
240 ns	(1) ALA364:O—ARG1:H2 (1.97 Å);<br>(2) GLN435:OE1—ALA4:H (2.05 Å);<br>(3) ARG438:HD1—CYS6:O (2.68 Å);<br>(4) TRP439:HD1—ARG1:O (2.67 Å);<br>(5) GLN326:OE1—ARG1:HA (2.69 Å);	(1) ALA434 (4.45 Å);<br>(2) ARG436 (4.95 Å);<br>(3) ARG438 (4.68 Å);<br>(4) TYR22 (4.69 Å);<br>(5) HIS157 (5.28 Å);

	(6) ASP182:OD2—ARG1:HD1 (2.73 Å);	(6) TRP439 (4.80 Å);
	(7) GLN435:OE1—THR3:HA (2.27 Å);	(7) TRP439 (5.36 Å);
	(8) GLN435:OE1—THR3:HB (2.99 Å);	
270 ns	(1) ARG438:HE—LEU8:OXT (1.93 Å);	(1) ALA434 (4.18 Å);
	(2) ALA364:O—ARG1:H1 (1.89 Å);	(2) ARG436 (4.75 Å);
	(3) GLN435:OE1—ALA4:H (1.93 Å);	(3) ARG438 (4.90 Å);
	(4) SER159:OG—LEU5:H (2.61 Å);	(4) HIS157 (5.04 Å);
	(5) ARG438:HD1—CYS6:O (2.41 Å);	(5) TRP439 (4.48 Å);
	(6) ASP182:OD2—ARG1:HD1 (2.65 Å);	(6) TRP439 (4.99 Å);
	(7) GLN435:OE1—THR3:HA (2.42 Å);	(7) TYR22 (4.82 Å);
300 ns	(1) ARG438:HE—LEU8:O (2.19 Å);	(1) ARG436 (4.65 Å);
	(2) ALA364:O—ARG1:H3 (2.01 Å);	(2) TYR22 (4.46 Å);
	(3) GLN435:OE1—ALA4:H (2.38 Å);	(3) HIS157 (4.75 Å);
	(4) SER159:OG—LEU5:H (2.16 Å);	(4) ARG438 (4.78 Å);
	(5) ASP182:OD2—ARG1:HD1 (2.66 Å);	(5) TRP439 (4.57 Å);
	(6) ASP182:OD2—ARG1:HD2 (2.81 Å);	(6) TRP439 (4.42 Å);
	(7) GLN435:OE1—THR3:HA (2.43 Å);	(7) TRP439 (2.53 Å);
	(8) GLN435:OE1—THR3:HB (2.52 Å);	
	(9) ARG438:HD1—CYS6:O (2.66 Å);	

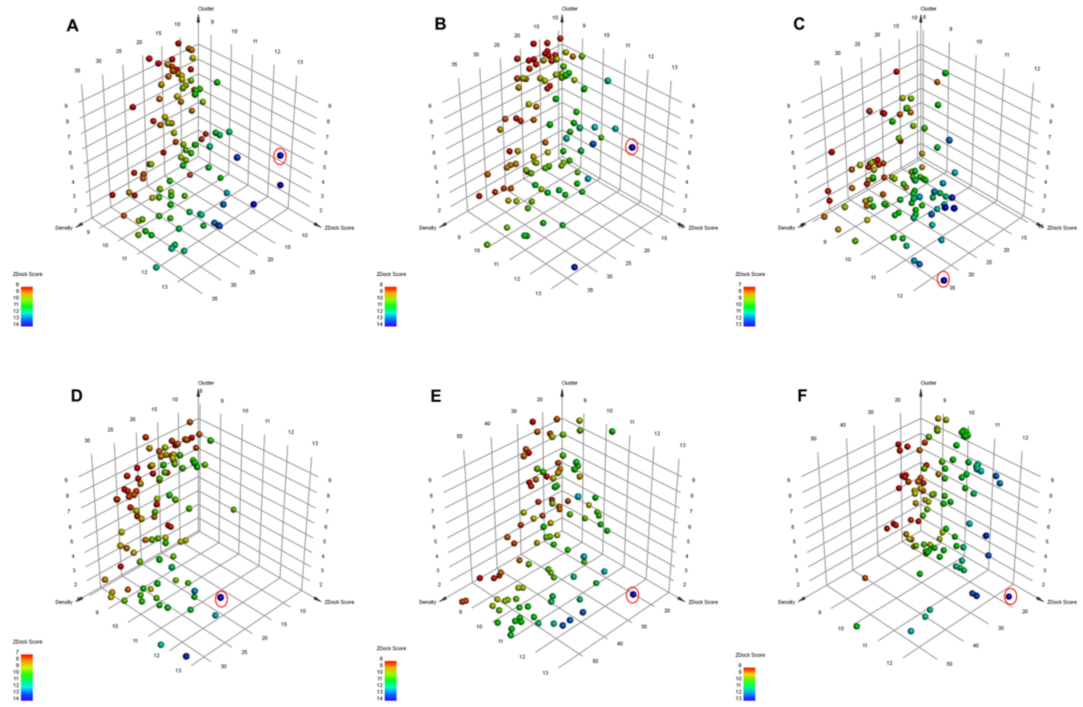

Figure S1. ZDOCK scores of top ranked poses and the chosen one. A. PvCesA2-NoPv1. B. PvCesA2-NoPv2. C. PvCesA2-NoPv3. D. PvCesA2-NoPv1R1A. E. PvCesA2-NoPv1R1AR7A. F. PvCesA2-NoPv1R7A.

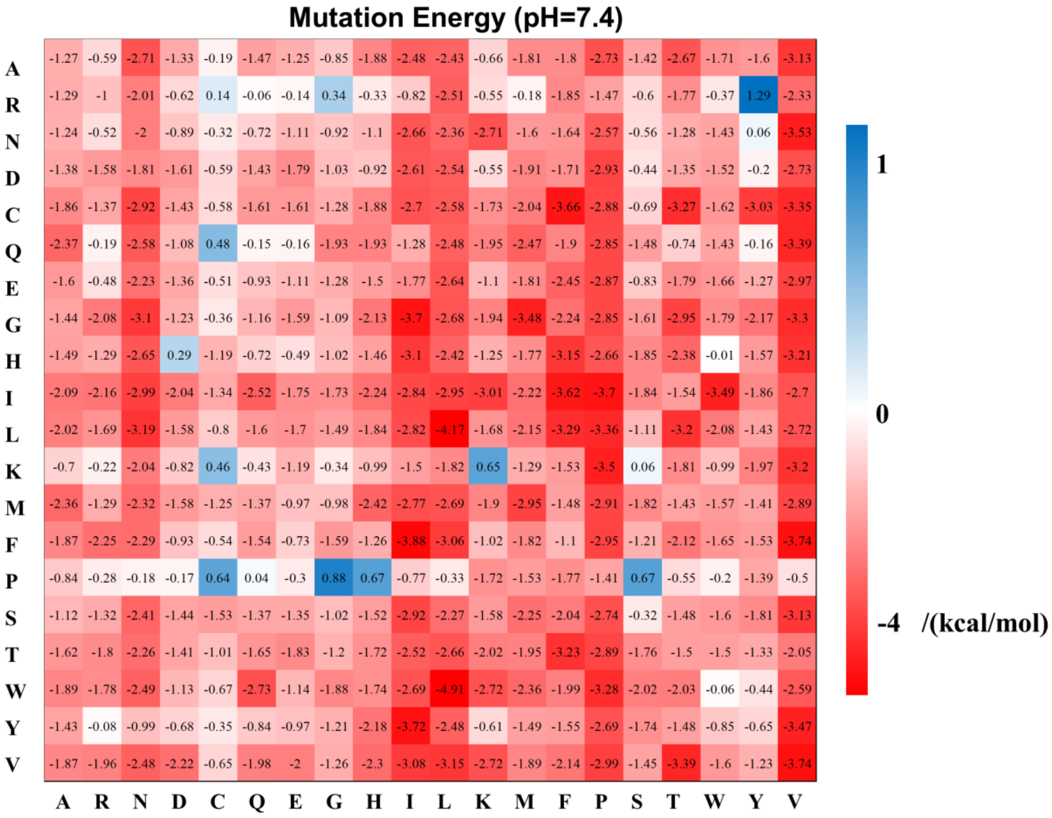

Figure S2. Predicted combined mutation energy of PvCesA2-NoPv1 binding at pH 7.4.

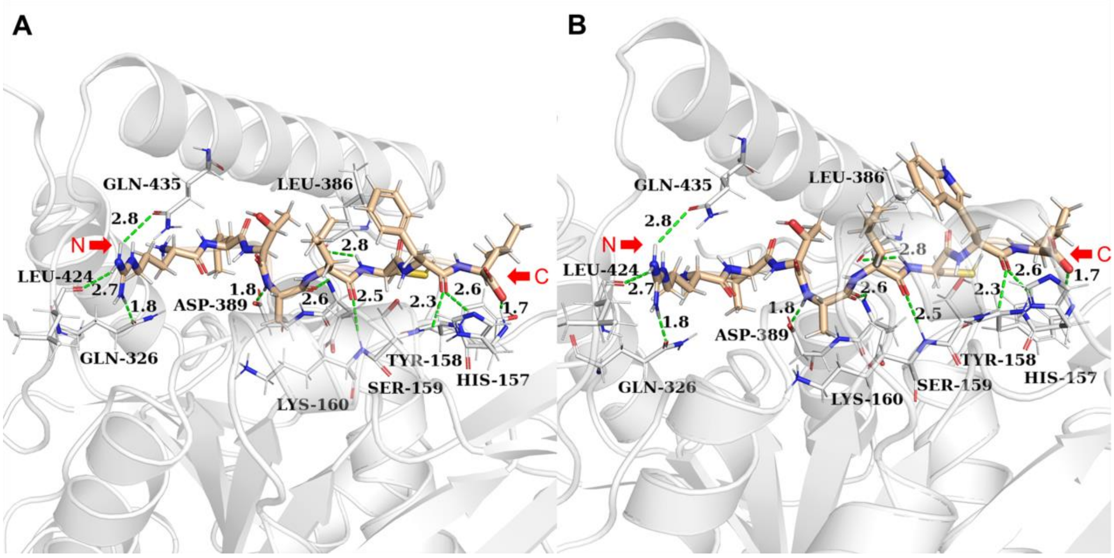

Figure S3. Interaction of mutants of NoPv1 with PvCesA2 after saturation mutations. A. Interaction between DP1 and PvCesA2. B. Interaction between DP2 and PvCesA2. The green dashed lines represent conventional hydrogen bonds.

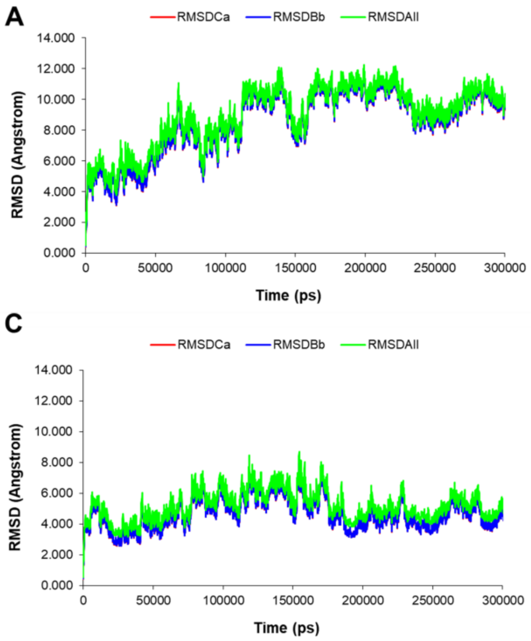

Figure S4. The RMSD profiles of 300 ns MD simulations of complexes and protein. A. PvCesA2 and NoPv1. B. PvCesA2 and DP1. C. PvCesA2 and DP2. D. PvCesA2. The RMSD values of Calpha [RMSDCa], backbone [RMSDBb], and all heavy atoms [RMSDAll] are shown in red, blue, and green, respectively.

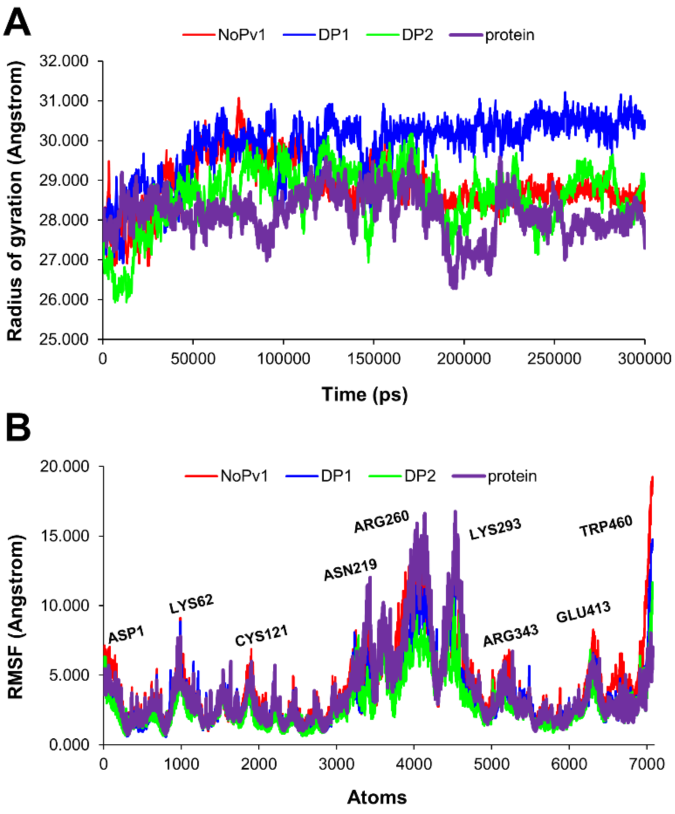

Figure S5. The relevant analysis of 300 ns MD simulations of complexes and protein. A. The plot of radius of gyration of complexes and protein at 300 ns simulation time. B. The RMSF values of complexes and protein at 300 ns simulation. The parameters of NoPv1, DP1, DP2, and protein are shown in red, blue, green, and purple, respectively.

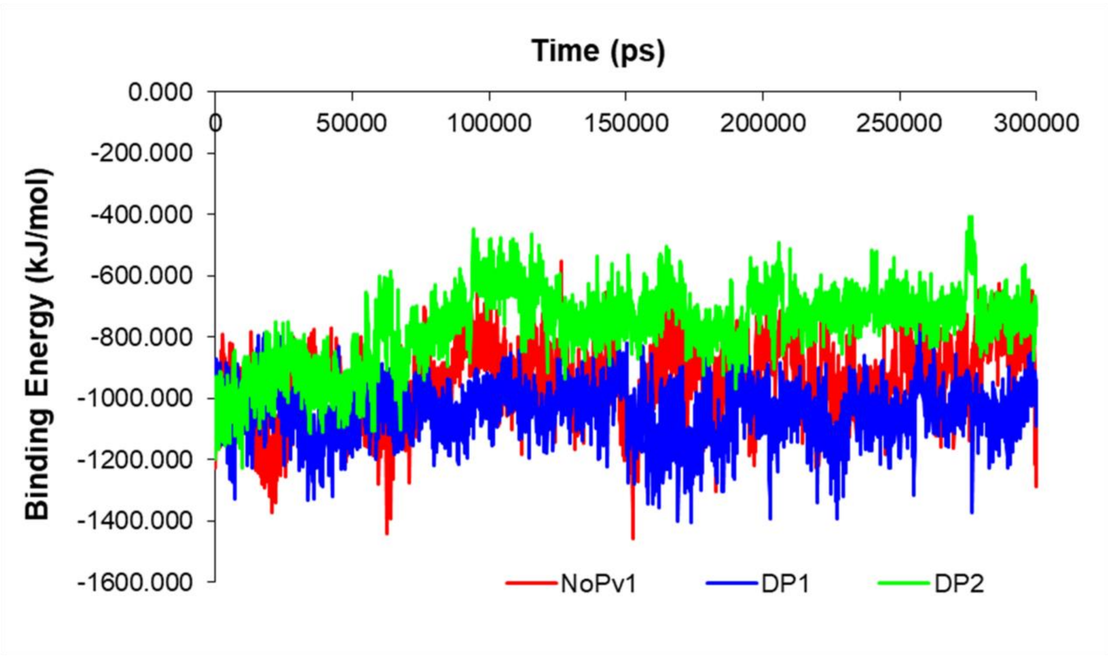

Figure S6. The Binding Energy profiles of 300 ns MD simulations of PvCesA2 with NoPv1/ DP1/DP2. The Binding Energy values of NoPv1, DP1, and DP2 are shown in red, blue, and green, respectively.

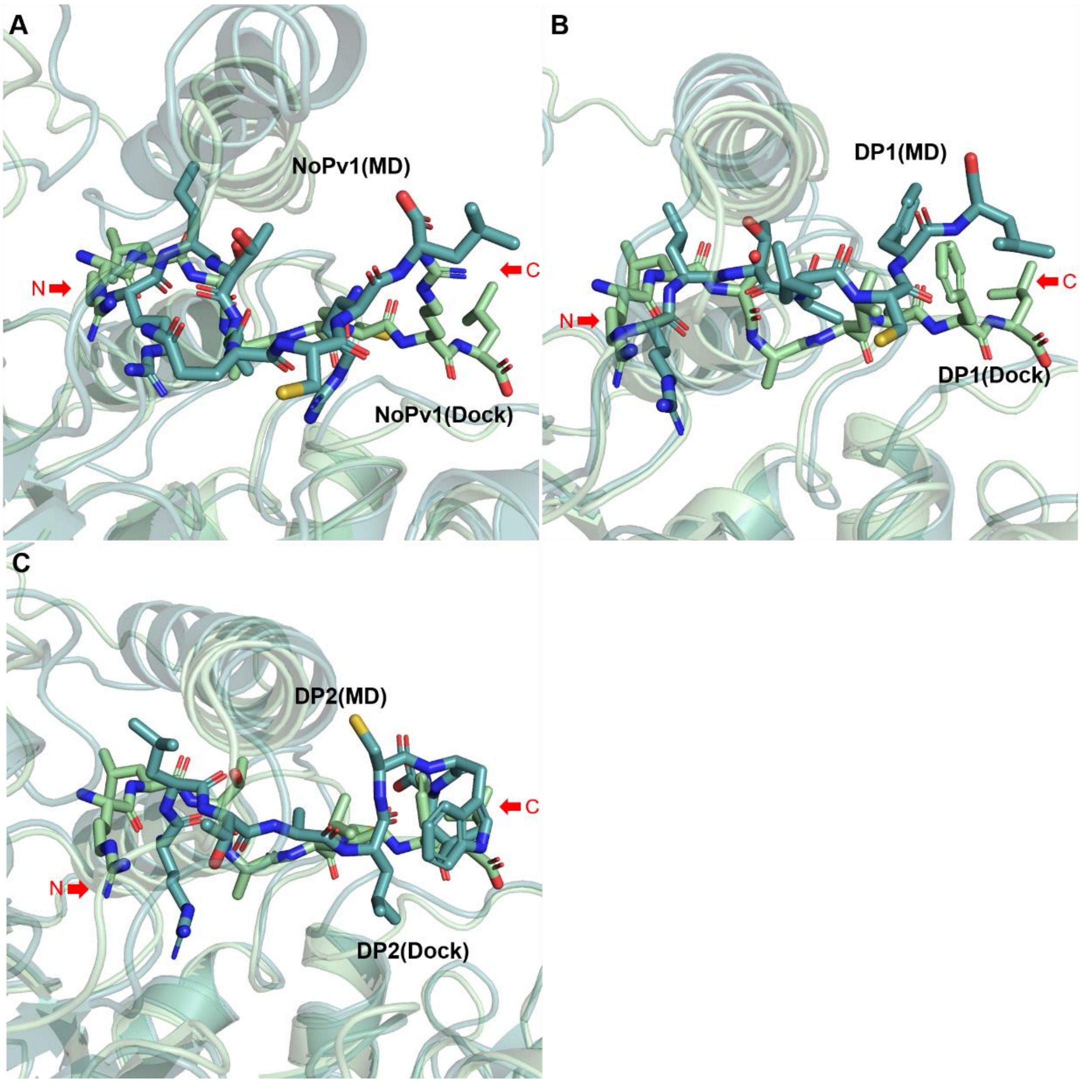

Figure S7. Superposition of two complexes including the last structures obtained from the MD and the complexes obtained from docking. The RMSD values of the structures aligned by PyMOL including the last structure from the MD (colored in light teal) and the complexes obtained from docking (colored in pale green) are 2.015, 1.867, and 1.981, respectively. A. PvCesA2-NoPv1. B. PvCesA2-DP1. C. PvCesA2-DP2.

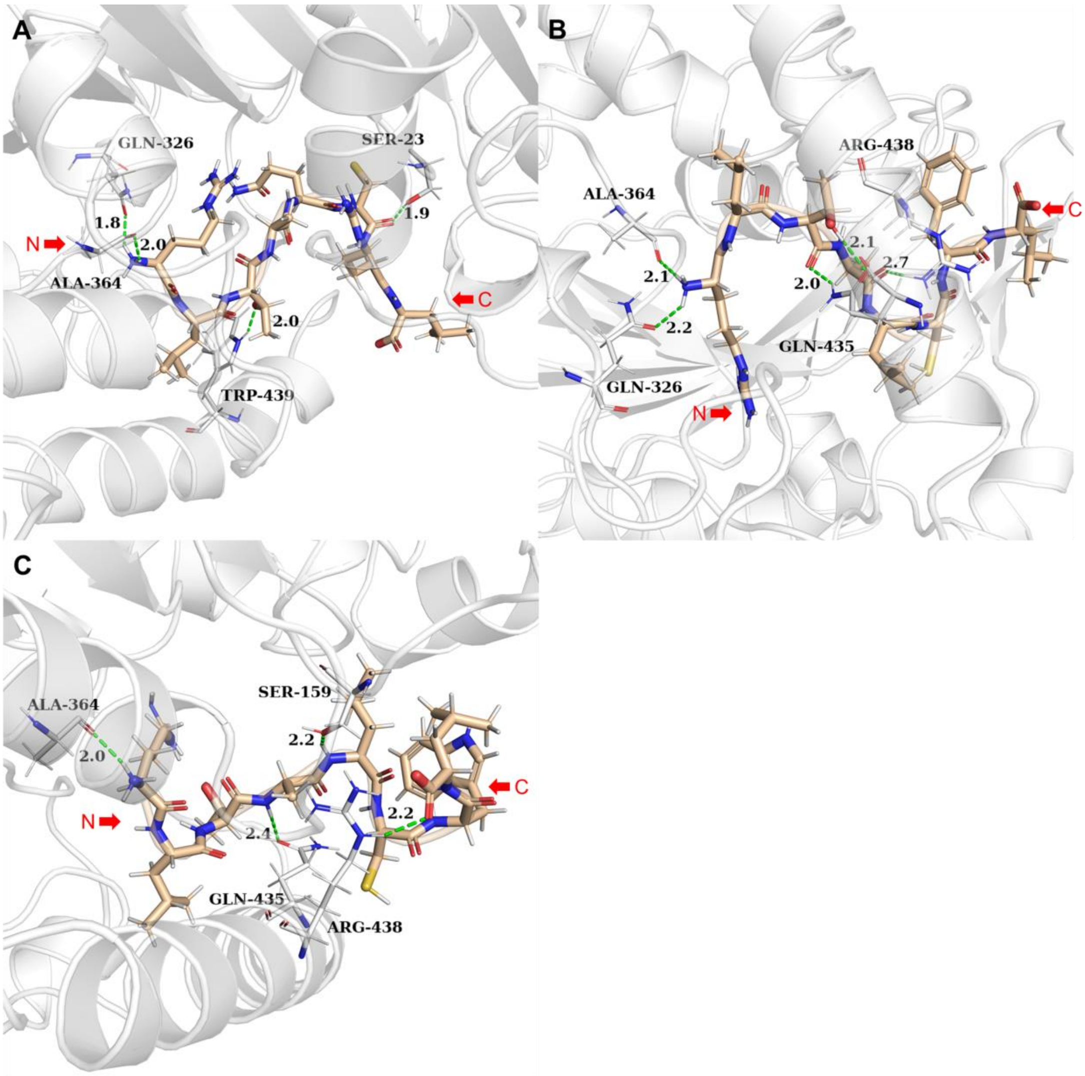

Figure S8. Interactions of the final structures obtained from the MD. A. Interaction between NoPv1 and PvCesA2. B. Interaction between DP1 and PvCesA2. C. Interaction between DP2 and PvCesA2. The green dashed lines represent conventional hydrogen bonds.
